# The mechanotransduction protein STOML3 is required for proprioceptor plasticity following peripheral nerve regeneration

**DOI:** 10.1113/EP092428

**Published:** 2025-03-31

**Authors:** Julia Haseleu, Jan Walcher, Gary R. Lewin

**Affiliations:** ^1^ Department of Neuroscience Max Delbrück Center for Molecular Medicine Berlin Germany; ^2^ Charité‐Universitätsmedizin Berlin, Charitéplatz 1 Berlin Germany; ^3^ German Center for Mental Health (DZPG), partner site Berlin Berlin Germany

**Keywords:** ion channel, mechanotransduction, peripheral nerve, proprioceptor, regeneration, spinal cord plasticity

## Abstract

Nerve regeneration is associated with the plasticity of sensory neurons such that even muscle afferents directed to the skin form mechanosensitive receptive fields appropriate for the new target. STOML3 is an essential mechanotransduction component in many cutaneous mechanoreceptors. Here, we asked whether STOML3 is required for functional and anatomical plasticity following peripheral nerve regeneration. We used a cross‐anastomosis model adapted to the mouse, in which the medial gastrocnemius nerve was redirected to innervate hairy skin previously occupied by the sural nerve. We recorded from muscle afferents innervating the skin and found that in wild‐type mice their receptive properties were largely identical to normal skin mechanoreceptors. However, in mice lacking STOML3, muscle afferents largely failed to form functional mechanosensitive receptive fields, despite making anatomically appropriate endings in the skin. Our tracing experiments demonstrated that muscle afferents from both wild‐type and *stoml3* mutant mice display remarkable anatomical plasticity, forming new somatotopically appropriate synaptic terminals in the region of the dorsal horn representing the sural nerve territory. The dramatic reduction in stimulus‐evoked activity from the cross‐anastomosed gastrocnemius nerve in *stoml3* mutant mice did not prevent central anatomical plasticity. Our results have identified a molecular factor required for functional plasticity following peripheral nerve injury.

## Abstract (highlights)

- **What is the central question of this study?**
  Does sensory mechanotransduction play a role in the functional recovery of regenerating axons when they reinnervate their original target or a new target after axonal injury?
- **What is the main finding and its importance?**
  We identify the mechanotransduction protein STOML3 as being necessary for muscle afferents, including proprioceptors, to make functional receptive fields in a new target. This is the first demonstration of a molecule required specifically for mechanoreceptor plasticity in vivo.

## 1 INTRODUCTION

Sensory processing relies fundamentally on topographic mapping. Sensory inputs are spatially segregated and tiled to construct a somatotopic map within the dorsal horn that defines the location of the stimulus (Shortland & Woolf, 1993; Shortland et al., 1989). After peripheral nerve transection and repair, most sensory axons can reach their target end‐organs (Fawcett & Keynes, 1990; Tedeschi & Bradke, 2017), but do not regain their original topographical position in the skin (Burgess & Horch, 1973; Horch, 1979; Koerber et al., 1989; Lewin et al., 1994). Consequently, cutaneous receptive fields of dorsal horn neurons receiving input from regenerated fibres are initially large and diffuse (Lewin et al., 1994), but after regeneration is complete the receptive fields shrink in an activity‐dependent manner (Lewin et al., 1994). Since the pioneering work of Rivers and Head (1908) it has been known that regenerated sensory neurons form functional mechanosensory receptive fields (Burgess & Horch, 1973; Dykes & Terzis, 1979; Terzis & Dykes, 1980).

There has been much progress in investigating the molecular factors, both intrinsic and extrinsic, that drive regeneration of sensory axons (Chen et al., 2007; Mahar & Cavalli, 2018; Tedeschi & Bradke, 2017). For example, hypoxia‐inducible factor‐1a was identified as a factor that can increase axon growth (Cho et al., 2015), but few studies have investigated how mechanosensitivity of regenerated axons is restored. Within hours after axotomy, severed axons become mechanosensitive (Koschorke et al., 1994), a finding indicating that mechanosensitive ion channels are already present in regenerating sensory axons. Recently, the first molecular components of the sensory mechanotransduction apparatus have been identified in sensory neurons (Chakrabarti et al., 2024; Poole et al., 2014; Ranade et al., 2014; Wetzel et al., 2007). One of these molecules is the integral membrane protein stomatin‐like protein‐3 (STOML3), which is an essential component of the mechanotransduction complex in many mechanoreceptors (Wetzel et al., 2007, 2017) and works by dramatically increasing the sensitivity of mechanosensitive PIEZO2 channels and can also regulate ELKIN1 ion channels (Chakrabarti et al., 2024; Poole et al., 2014). The *PIEZO2* gene is required for normal touch sensation in humans (Chesler et al., 2016), and many mechanoreceptors and proprioceptors need this protein to respond to mechanical stimuli in mice (Ranade et al., 2014; Woo et al., 2015). Indeed, in either *stoml3* or *Piezo2* mutant mice ∼40% of cutaneous myelinated sensory afferents completely lack mechanosensitivity (Ranade et al., 2014; Wetzel et al., 2007, 2017). In this study, we have addressed two related questions. First, does sensory mechanotransduction play a role in the functional recovery of regenerating axons when they reinnervate their original target or a new target after axonal injury? Second, does activity driven by mechanical stimulation play a crucial role in central plasticity after axonal injury?

We used a cross‐anastomosis model in which the gastrocnemius nerve, a pure muscle nerve, is forced to regrow into the cutaneous sural nerve territory (McMahon & Gibson, 1987; McMahon & Wall, 1989). In this model, muscle afferents including proprioceptors are capable of functionally innervating foreign targets and gaining receptive field properties appropriate for the new cutaneous target (Johnson et al., 1995; Lewin & McMahon, 1991a, 1991b, 1993). Muscle afferents that are forced to innervate the skin display substantial plasticity, making new functional connections in the spinal cord appropriate for the new target (Lewin & McMahon, 1993; McMahon & Wall, 1989). Here, we established this model in the mouse, which allowed us to ask whether normal functional recovery after nerve regeneration requires the presence of the mechanotransduction molecule STOML3. Surprisingly, we found that STOML3 is required for most muscle afferents to make mechanosensitive endings in the skin. However, substantial central plasticity of the central terminals of these muscle afferents was still observed in the spinal cord. Our findings identify, for the first time, a molecular factor crucial for the functional recovery of regenerating axons in the adult peripheral nervous system.

## 2 MATERIALS AND METHODS

### 2.1 Ethical approval

Mice used in this study were *stoml3* mutant mice (Wetzel et al., 2007, 2017), which were bred over >10 generations onto a C57BL/6 background. The same C57BL/6 strain used for backcrossing was used as a control. All regulated procedures carried out on animals involved in this publication were applied for and approved by the Landesamt für Gesundheit und Soziales (LaGeSo, State of Berlin) and were in full compliance with German and EU animal protection laws (reference G0239/11). Tissues for physiological and anatomical experiments were obtained from animals that were killed humanely by cervical dislocation or by exposure to a rising concentration of CO_2_ gas. Before and throughout the experiments, mice were maintained in plastic cages with commercial bedding, tap water and commercial pelleted diet freely provided and kept in rooms with a 12 h–12 h light–dark cycle. All mice were housed and handled according to the German Animal Protection Law.

### 2.2 Transganglionic tracing with cholera toxin subunit B conjugates

Four‐ to 5‐week‐old female and male mice were anesthetized by an intraperitoneal injection of ketamine (100 mg/kg) and xylazine (10 mg/kg) (Sigma‐Aldrich). They were injected subcutaneously with 0.2 µL of 1.5% cholera toxin subunit B conjugated with Alexa Fluor 594 (CTB; Thermo Fisher Scientific, Waltham, MA, USA) into the second digit of the left hind paw and the third digit of the right hind paw, using a pulled glass pipette (5 µL PCR Pipets, Drummond Scientific Co., Broomall, PA, USA) attached to a Hamilton microlitre syringe (Hamilton Bonaduz AG, Bonaduz, Switzerland). The glass capillary was inserted into the most distal interphalangeal crease and advanced under the skin towards the next proximal crease, where the tracer was injected slowly. Five days postinjection, allowing for transganglionic transport of the tracer, the anesthetized mice were perfused transcardially with 0.1 M PBS and ice‐cold 4% paraformaldehyde (PFA). Subsequently, tissues of interest [lumbar dorsal root ganglia (DRGs), spinal cord and hind paw skin] were dissected out and postfixed overnight in 4% PFA at 4°C.

Mice subjected to cross‐anastomosis surgeries were anaesthetized 12 weeks postsurgery as described above. For intraneural CTB injections, the sural nerve was exposed in the popliteal fossa by an incision of the biceps femoris. To enable the insertion of a glass capillary, the nerve was freed from surrounding tissue and carefully lifted up by placing a small spatula under it. After puncturing its epineurium, the tip of a pulled glass capillary that was attached to a Hamilton microlitre syringe was carefully inserted into the nerve, and 2 µL of 1.5% CTB in 0.1 M PBS was injected slowly. Subsequently, the wound was washed out with 0.1 M PBS and closed using sterile sutures. The tracer was allowed to be transported transganglionically for 5 days, after which the mice were perfused transcardially. Tissues of interest (spinal cord and peripheral nerves) were dissected out and postfixed overnight in 4% PFA at 4°C.

### 2.3 Cross‐anastomosis surgery

Four‐ to 5‐week‐old female and male mice were anaesthetized by an intraperitoneal injection of ketamine (100 mg/kg) and xylazine (10 mg/kg). We chose to use relatively young mice because of the long regeneration times used in the experiment, meaning that the mice were mature adults when final experiments were carried out. The sural nerve and the medial gastrocnemius nerve were exposed in the popliteal fossa by an incision of the biceps femoris, cut and cross‐anastomosed as described before (Lewin & McMahon, 1991a; McMahon & Gibson, 1987). Briefly, the proximal stump of the sural nerve was joined to the distal stump of the medial gastrocnemius nerve and vice versa with an epineural suture stitch using swaged microsurgical sutures (11/0). On the contralateral side, the two nerves were left intact, transected or self‐anastomosed. The wounds were washed out with 0.1 M PBS and closed in layers using sterile sutures. Animals were monitored closely postsurgery and endpoints were defined for euthanasia (>20% weight loss, walking difficulty, walking on tip‐toe or coma within the first 48 h). Every animal in the study recovered from surgery without meeting any of the above outlined endpoint criteria. In each case, animals were given analgesics for 3 days following surgery (carprofen 5 mg/kg subcutaneously). After 12 weeks, the mice were either killed in order to perform skin–nerve preparation experiments or subjected to transganglionic tracing experiments.

### 2.4 Tissue clearing

Routinely, fixed spinal cords were washed three times with 0.1 M PBS for 10 min each at room temperature (RT). Subsequently, they were immersed in an ascending concentration series of 2,2′‐thiodiethanol (TDE) for 24 h each at RT (Aoyagi et al., 2015; Costantini et al., 2015; Kloepper et al., 2010; Staudt et al., 2007). The applied concentrations were 10%, 25%, 50% and 97% TDE diluted with 0.1 M PBS. Alternatively, spinal cords were cleared using the optical clearing technique three‐dimensional imaging of solvent‐cleared organs (3DISCO), which is based on tetrahydrofuran (THF) and dibenzyl ether (DBE) or a combination of THF and TDE (Becker et al., 2013; Ertürk et al., 2012, 2011). Briefly, spinal cords were washed three times with 0.1 M PBS for 10 min each at RT. Subsequently, they were dehydrated and delipidated in 50%, 70% and 80% THF diluted with ddH_2_O for 30 min each and three times in 100% THF for 30 min at RT. Then, the dehydrated tissues were either immersed in 100% DBE or in an ascending concentration series of TDE.

Fixed whole‐mount skin samples were washed three times with 0.1 M PBS for 10 min each at RT. Subsequently, the tissues were dehydrated and delipidated in 50%, 70% and 80% THF diluted with ddH_2_O for 30 min each and three times in 100% THF for 30 min at RT. Finally, the dehydrated specimens were incubated in an ascending concentration series of TDE for 12 h each at RT.

During all incubation steps, the samples were kept on a vibrating table in the dark. For imaging, cleared spinal cords and whole‐mount skin samples were mounted on glass slides in 97% TDE using press‐to‐seal silicone isolators (Electron Microscopy Sciences, Hatfield, PA, USA), with the dorsal surface of the spinal cords facing up.

Fixed DRGs, peripheral nerves and immunostained skin slices were cleared in an ascending concentration series of TDE for a minimum of 120 min per concentration step and mounted on glass slides with coverslips.

### 2.5 Immunostaining of thick skin slices

Dissected skin was postfixed in 4% PFA overnight. Subsequently, the skin was washed three times in PBS at RT for 10 min each and embedded in 3% low‐melting‐point agarose. Using a vibratome, the skin was cut into 100‐µm‐thick transverse slices. Prior to antibody incubation, the skin slices were washed in blocking solution (5% normal serum and 0.1% Triton X‐100 in 0.1 M PBS) at 4°C for 1 h. Skin slices were incubated with primary antibodies diluted in a blocking solution at 4°C for 24 h. Next, the slices were washed three times in 0.1 M PBS for 10 min each at RT and incubated with secondary antibodies diluted in a blocking solution at 4°C for 24 h. All incubation steps were performed under agitation and in the dark. After completion of immunostaining, skin slices were optically cleared. Primary antibodies used were as follows: chicken antiNF200 (Abcam, catalogue no. ab72996, RRID: AB_2149618) 1:2000; rabbit antiPGP9.5 (Dako, catalogue no. Z5116, RRID: AB_2622233) 1:500; rabbit antiS100 (Dako, catalogue no. Z0311, RRID: AB_10013383) 1:1000; and rat antiCytokeratin8/18 (TROMA‐I; DSHB, catalogue no. TROMA‐1, RRID: AB_531826) 1:1000. Secondary antibodies (Invitrogen) were coupled to Alexa Fluor dyes (488 or 647) and used at a dilution of 1:1000.

### 2.6 Two‐photon microscopy

Two‐photon imaging was performed using a laser scanning microscope (LSM710 NLO; Carl Zeiss, Oberkochen, Germany) equipped with a tunable Ti:sapphire laser (Chameleon; Coherent, Santa Clara, CA, USA). Two channels were recorded sequentially to collect Alexa Fluor 594 fluorescence (excitation wavelength, 840 nm; emission range, 589–735 nm) and tissue autofluorescence (excitation wavelength, 780 nm; emission range, 504–608 nm). A ×25 multi‐immersion objective (0.8 numerical aperture) was used with water for uncleared sample imaging and with immersion oil for cleared sample imaging. Tiled stacks were taken through the spinal cord dorsal horn (for images shown in Figure 2, pixel size was 0.55 µm × 0.55 µm and *z*‐step size was 3 µm; for images shown in Figure 6, pixel size was 1 µm × 1 µm and *z*‐step size was 2 µm) and DRGs (pixel size, 1 µm × 1 µm and *z*‐step size, 4 µm).

### 2.7 Confocal microscopy

Cleared whole‐mount and sectioned skin samples were imaged using a laser scanning microscope (Zeiss LSM 710 NLO, Carl Zeiss) equipped with a ×10 objective (0.3 numerical aperture) and a ×25 objective (0.8 numerical aperture). Fluorescence and transillumination images were acquired simultaneously.

### 2.8 Electron microscopy

For electron microscopy, mice were perfused transcardially with 0.1 M PBS and ice‐cold 4% PFA. Nerves were dissected and postfixed in 4% PFA and 2.5% glutaraldehyde in 0.1 M PBS for 3 days. Following treatment with 1% OsO_4_ in 0.1 M PBS for 2 h at RT, the nerves were washed two times in 0.1 M PBS, dehydrated in a graded ethanol series and propylene oxide, and embedded in Poly/Bed 812 (Polysciences Europe GmbH, Hirschberg an der Bergstraße, Germany). Semithin sections were stained with Toluidine Blue. Ultrathin sections (70 nm) were contrasted with uranyl acetate and lead citrate. Sections were examined with a Zeiss 910 electron microscope (Carl Zeiss), and digital images were taken with a high‐speed slow‐scan CCD camera (Proscan, Lagerlechfeld, Germany) at an original magnification of ×1600. Three ultrathin sections were taken from three nerves, and on each ultrathin section five images (16.83 µm × 12.91 µm) were taken. Myelinated axons were counted in these areas using ImageJ (Schneider et al., 2012). Axon counts were normalized to the whole nerve.

### 2.9 Image processing

All images were processed using ImageJ (Schneider et al., 2012). Tiled stacks were stitched using either the imaging software ZEN 2010 (Carl Zeiss) or the ImageJ plugin ‘Stitching 2D/3D’ (Preibisch et al., 2009). Subsequently, images were cropped to the same size and reduced to the same number of slices. Next, background fluorescence was reduced by subtracting the autofluorescence channel from the CTB channel. Using stack histogram‐based thresholding, the image stacks were binarized. The threshold was set as the mean grey value plus three times the SD. Finally, single pixels were removed to reduce noise, such as hot pixels (Video S1).

In order to enable comparative analyses of spinal terminal fields of cutaneous myelinated afferents, the three‐dimensional centres of mass of the voxel clouds representing CTB‐labelled fibre terminals were determined using the ImageJ plugin ‘3D ImageJ Suite’ (Ollion et al., 2013). All images were aligned to the centre of mass of the voxel cloud, that is, images were cropped to the same size and reduced to the same number of slices around the respective centres of mass.

Summed dorsoventral, rostrocaudal and/or mediolateral projections of the binary image stacks were constructed to enable two‐dimensional visualization of terminal fields (Video S1).

### 2.10 Image analysis

Relative locations of spinal terminal field foci were determined with respect to the dorsal and medial grey–white matter border. The distance between the centre of mass of the terminal field and the dorsal and medial grey–white matter borders was measured in summed rostrocaudal and dorsoventral projections of binary image stacks, respectively.

Mediolateral, rostrocaudal and dorsoventral spans of the terminal fields were measured in summed dorsoventral (mediolateral and rostrocaudal spans) and rostrocaudal (dorsoventral span) projections of binary image stacks. Summed projections were thresholded, with the threshold being set as the mean grey value plus 1 SD. Subsequently, the dimensions of the bounding rectangles enclosing all pixels representing CTB‐labelled fibre terminals were measured.

Areal densities (as voxels per area) of spinal terminal fields were calculated in summed dorsoventral, mediolateral and rostrocaudal projections of binary image stacks. Using the ImageJ plugin ‘3D ImageJ Suite’, the total number of voxels representing CTB‐labelled fibre terminals was determined in binary image stacks. Subsequently, the number of voxels was divided by the area (in micrometres squared) that was occupied by positive pixels in summed dorsoventral, mediolateral and rostrocaudal projections, respectively.

### 2.11 *Ex vivo* skin–nerve preparation studies

*Ex vivo* skin–nerve preparations were performed as described before (Moshourab et al., 2013; Walcher et al., 2018). Briefly, mice were killed, and the hair on the left hindlimb was removed. The sural nerve (intact or regenerated) or the rerouted medial gastrocnemius nerve was exposed in the popliteal fossa and dissected free along the lower leg. Subsequently, the skin was carefully removed from the musculoskeletal and connective tissue of the paw. The skin–nerve preparation was placed in an organ bath filled with oxygenated 32°C warm synthetic interstitial fluid (mM; NaCl, 123; KCl, 3.5; MgSO_4_, 0.7; NaH_2_PO_4_, 1.7; CaCl_2_, 2.0; sodium gluconate, 9.5; glucose, 5.5; sucrose, 7.5; and HEPES, 10; at a pH of 7.4). Using insect needles, the skin was mounted in the organ bath with its epidermis facing the bottom of the chamber, exposing the dermis to the solution. The nerve was pulled through a hole into the adjacent recording chamber, which was filled with mineral oil. Finally, using fine forceps, the nerve was desheathed by removing its epineurium, and small filaments of the nerve were teased out. Throughout the whole experiment, the skin was superfused with oxygenated synthetic interstitial fluid at a flow rate of 15 mL/min.

Teased filaments were attached to a recording electrode. The receptive fields of individual units were identified by manually probing the skin with a glass rod. Subsequently, the units were classified as rapidly adapting and slowly adapting mechanoreceptors (RAMs and SAMs, respectively), in addition to A‐mechanonociceptors (AMs) or D‐hair receptors based on their conduction velocity (CV), spike pattern and sensitivity (Chakrabarti et al., 2024; Sánchez‐Carranza et al., 2024; Walcher et al., 2018). For immediate visual identification of single units, whole action potential waveforms were resolved on an oscilloscope. Data were acquired using a PowerLab 4/30 system (ADInstruments Ltd, Oxford, UK), which was controlled with the software LabChart v.7.1 (ADInstruments Ltd, Oxford, UK).

The CVs of single fibres were determined by evoking a local action potential with a platinum–iridium electrode (1 MΩ; World Precision Instruments Germany GmbH, Berlin, Germany). The electrical impulse was conducted almost instantaneously through the solution, whereas the triggered action potential conducted by the fibre was delayed depending on the fibre type and the distance of the receptive field of the fibre from the electrode. Hence, the distance between the receptive field of a unit and the electrode was measured, and the CV was calculated as distance divided by time delay. Routinely, fibres with CVs of >10 m/s were classified as Aβ‐fibres and those with CVs between 1.5 and 10 m/s were classified as Aδ‐fibres.

Mechanically sensitive receptors were stimulated using either a piezo actuator (Physik Instrumente GmbH & Co. KG, Karlsruhe Germany) delivering dynamic and vibratory stimuli or a nanomotor (Kleindieck Nanotechnik GmbH, Reutlingen, Germany) enabling static stimulations. Both the piezo actuator and the nanomotor were connected to a force sensor and mounted on a manual micromanipulator. Based on their response properties to various ramp‐and‐hold stimuli, Aβ‐ and Aδ‐fibres were classified further as RAMs or SAMs and D‐hair receptors or AMs, respectively. Using the piezo actuator, dynamic mechanical stimuli were delivered in the form of ramp‐and‐hold stimuli with constant force (∼40 mN) but ramp phases of different velocities (0.075, 0.15, 0.45 and 1.5 mm/s). Spikes elicited during the dynamic phase of the stimulus were analysed. Furthermore, sinusoidal vibration stimuli (25 and 50 Hz) increasing in amplitude were given to determine the mechanical threshold of the fibre as the minimal force needed to evoke an action potential. Static mechanical stimuli were delivered using the nanomotor, which was controlled by the NanoControl 4.0 software (Kleindieck Nanotechnik GmbH, Reutlingen, Germany). Ramp‐and‐hold stimuli with a constant ramp (1.5–2 mN/ms) but varying amplitudes were applied. Spikes evoked during the static phase of the stimulus were analysed.

For the electrical search protocol, a microelectrode (0.5–1 MΩ) was manoeuvred gently to contact the epineurium of the nerve, and electrical stimuli at 1 s intervals with square pulses of 50–500 ms duration were delivered. Electrically identified units were traced to their receptive fields. Subsequently, the mechanical sensitivity of single units was tested by mechanical stimulation of their receptive field with a glass rod; units not responding to mechanical probing were designated as mechano‐insensitive. Based on the CV, these units were categorized as mechano‐insensitive Aβ‐ or Aδ‐fibres.

### 2.12 Statistical analysis

All statistical analyses were performed using the statistical software Prism 6 (GraphPad Software Inc., La Jolla, CA, USA). Depending on the experimental design, datasets were analysed using Student's two‐tailed unpaired *t*‐test (with Welch's correction for datasets with unequal variances), one‐way ANOVA (with Tukey's multiple comparison test), two‐way repeated‐measures ANOVA (with Bonferroni's *post hoc* test) or two‐sided Fisher's exact tests. In cases where two‐way repeated‐measures ANOVA were performed, *P*‐values for interaction effects are stated. Datasets were considered significantly different for *P*‐values of <0.05. In figures, *P*‐values are represented using the following asterisk rating system: ^*^ *P* < 0.05, ^**^ *P* < 0.01 and ^***^ *P* < 0.001. All data are presented as the mean value ± SEM.

## 3 RESULTS

### 3.1 Structural and functional plasticity of primary sensory neurons

**FIGURE 1:**
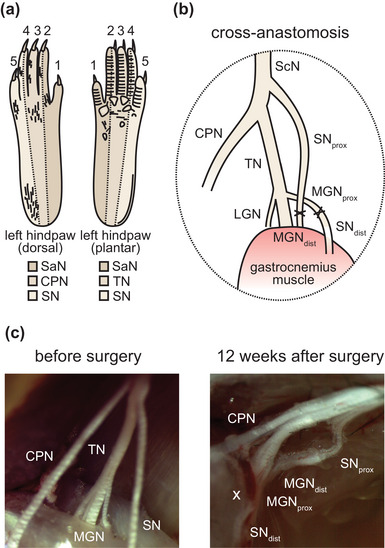
Cross‐anastomosis of the sural and gastrocnemius nerves in the popliteal fossa. (a) Schematic representation of the innervation territories of four peripheral nerves: saphenous nerve (SaN), common peroneal nerve (CPN), tibial nerve (TN) and sural nerve (SN) in the left hind paw. (b) In the cross‐anastomosis model, the sural nerve (SN) and the medial gastrocnemius nerve (MGN) are cross‐anastomosed. (c) Stereomicroscopic images of the peripheral nerves innervating the hind paw in the popliteal fossa before and 12 weeks after cross‐anastomosis surgery. The cross‐anastomosis sites are marked with an ‘X’. Abbreviations: CPN, common peroneal nerve; LGN, lateral gastrocnemius nerve; MGN, medial gastrocnemius nerve; SaN, saphenous nerve; ScN, sciatic nerve; SN, sural nerve; TN, tibial nerve.

We investigated how mechanosensory silence affects structural and functional plasticity following nerve injury. We adapted a cross‐anastomosis model to the mouse, in which the medial gastrocnemius nerve, a pure muscle nerve, is cross‐anastomosed to the cut cutaneous sural nerve, which innervates the lateral hind paw and ankle (McMahon & Wall, 1989; Lewin & McMahon, 1991a, 1991b). Initially, we performed cross‐anastomosis surgeries in wild‐type mice to investigate the capacity of muscle afferents to innervate the skin functionally (Figure 1a, b).

Cross‐anastomosis surgeries were performed on 4‐week‐old wild‐type mice. As a control, the sural nerve was either left intact or self‐anastomosed. During terminal experiments (12 weeks postsurgery), the cross‐anastomosed nerves were examined to exclude inappropriate nerve regeneration. In all cases, the cross‐anastomosed nerves showed intact epineural sheaths and were clearly separable from each other and from other peripheral nerves within the popliteal fossa (Figure 1c).

**FIGURE 2:**
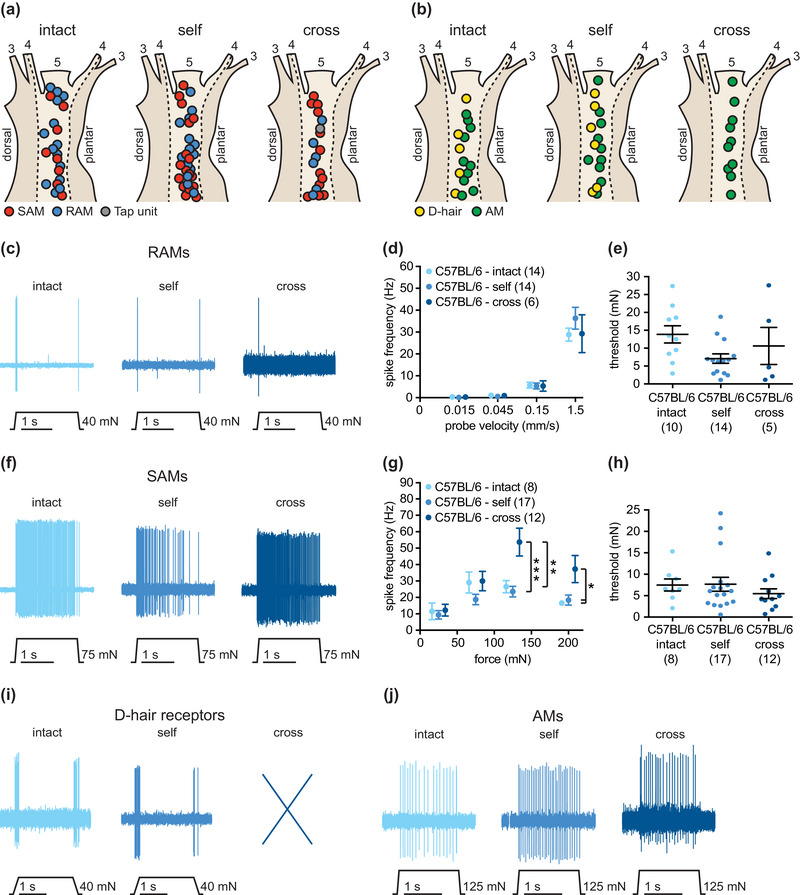
Response properties of muscle afferents newly innervating the skin in comparison to intact and regenerated cutaneous afferents in C57BL/6 mice. (a, b) Receptive field locations of intact cutaneous, regenerated cutaneous and redirected muscle afferent Aβ‐fibres (a; SAMs, red; RAMs, blue; tap‐units, grey) and Aδ‐fibres (b; D‐hair receptors, yellow; AMs, green). (c) Example traces of RAM responses to a ramp‐and‐hold stimulus with a probe velocity of 1.5 mm/s. (d) Spike frequencies of RAMs in response to ramp‐and‐hold stimuli with increasing ramp velocities. Mean values ± SEM. (e) Mechanical thresholds of RAMs measured in response to a sinusoidal vibration stimulus (50 Hz). Individual data points and mean values ± SEM are shown for spike responses to ramp stimuli [‘intact’, *n* = 10 fibres; ‘self’, *n* = 14 fibres; ‘cross’, *n* = 5 fibres; two‐way repeated‐measures ANOVA: *F*(6, 93) = 0.9800, *P* = 0.443 and for mechanical thresholds [‘intact’, 13.9 ± 2.4 mN, *n* = 10 fibres; ‘self’, 7.1 ± 1.3 mN, *n* = 14 fibres; ‘cross’, 10.6 ± 5.2 mN, *n* = 5 fibres; one‐way ANOVA: *F*(2, 26) = 2.543, *P* = 0.098]. (f) Example traces of SAM responses to a ramp‐and‐hold stimulus with an indentation force of 75 mN. (g) Spike frequencies of SAMs in response to a series of increasing displacement stimuli. Note that cross‐anastomosed gastrocnemius SAMs showed enhanced stimulus responses in comparison to intact and self‐anastomosed nerves [‘intact’, *n* = 8 fibres; ‘self’, *n* = 17 fibres; ‘cross’, *n* = 12 fibres; two‐way repeated‐measures ANOVA: *F*(6, 102) = 4.700, *P* < 0.001; Bonferroni's *post hoc* test]. Mean values ± SEM are shown. (h) Mechanical thresholds, the minimum force needed to evoke an action potential, of SAMs. Individual data points and mean values ± SEM are shown. No significant differences were noted [‘intact’, 7.5 ± 1.4 mN, *n* = 8 fibres; ‘self’, 7.7 ± 1.6 mN, *n* = 17 fibres; ‘cross’, 5.5 ± 1.1 mN, *n* = 12 fibres; one‐way ANOVA: *F*(2, 34) = 0.6402, *P* = 0.533]. (i) Example traces of D‐hair receptor responses to a ramp‐and‐hold stimulus with a probe velocity of 0.45 mm/s. (j) Example traces of AM responses to a ramp‐and‐hold stimulus with an indentation force of 125 mN. Abbreviations: AM, A‐mechanonociceptor; D‐hair, Down‐hair; RAM, rapidly adapting mechanoreceptor; SAM, slowly adapting mechanoreceptor.

We made extracellular recordings from single intact and regenerated fibres in wild‐type mice using the *ex vivo* skin–nerve preparation adapted to the sural nerve territory. Consistent with previous studies in the cat and rat (Johnson et al., 1995; Lewin & McMahon, 1991a), single units with response properties characteristic of rapidly adapting and slowly adapting mechanoreceptors (RAMs and SAMs) and AMs were found in all three preparations (‘intact’, ‘self’ and ‘cross’) (Figure 2a,b). Functional D‐hair receptors were found only in preparations of the intact and regenerated sural nerve, but not in preparations where muscle afferents were redirected towards the skin (Figure 2b). In the cross‐anastomosed gastrocnemius nerve, one Aβ‐fibre (1 of 18) was found to respond only to manually delivered rapid and vigorous tapping of the receptive field, a rapid change in force beyond what could be delivered by the electromechanical stimulator (>1.5 mm/s) (Figure 2a). This type of mechanically insensitive fibre type (tap‐unit) has been noted rarely in wild‐type nerves, but was found to be more frequent in *stoml3* mutant mice (Moshourab et al., 2013; Wetzel et al., 2007). Table 1 provides an overview of the number of units characterized in the three preparations and their conduction velocities. As expected from the results of previous studies (Horch & Lisney, 1981; Johnson et al., 1995; Lewin & McMahon, 1991a), the conduction velocities of Aβ‐fibres were significantly slower in the regenerated nerves (both self‐ and cross‐anastomosed nerves) when compared with those recorded from the intact nerve (Table 1). In contrast, the conduction velocities of Aδ‐fibres were not significantly different between any of the three experimental groups (Table 1).

**TABLE 1:** Conduction velocities of intact cutaneous, regenerated cutaneous and redirected muscle afferents innervating hind paw skin in C57BL/6 and *stoml3* mutant mice.

C57BL/6	Intact (*n* = 6 mice)	Self (*n* = 10 mice)	Cross (*n* = 9 mice)	Statistics
Aβ‐fibres CV (m/s)	*n* = 22 15.58 ± 0.72	*n* = 31 11.84 ± 0.37	*n* = 18 10.82 ± 0.50	*F*(2, 68) = 20.66 *P* < 0.0001
SAMs CV (m/s)	*n* = 8 17.09 ± 1.19	*n* = 17 11.69 ± 0.48	*n* = 12 11.00 ± 0.63	*F*(2, 34) = 18.09 *P* < 0.0001
RAMs CV (m/s)	*n* = 14 14.72 ± 0.86	*n* = 14 12.04 ± 0.58	*n* = 6 10.45 ± 0.85	*F*(2, 31) = 6.592 *P* = 0.0041
Aδ‐fibres CV (m/s)	*n* = 15 5.14 ± 0.37	*n* = 16 4.48 ± 0.50	*n* = 10 3.55 ± 0.70	*F*(2, 38) = 2.22 *P* = 0.1225
AMs CV (m/s)	*n* = 10 5.18 ± 0.53	*n* = 10 3.61 ± 0.59	*n* = 10 3.55 ± 0.69	*F*(2, 27) = 2.287 *P* = 0.1209
D‐hairs CV (m/s)	*n* = 5 5.06 ± 0.38	*n* = 6 5.92 ± 0.45	*n* = 0	*P* = 0.1896

*stoml3* ^−/−^	Intact (*n* = 5 mice)	Self (*n* = 6 mice)	Cross (*n* = 10 mice)	Statistics
Aβ‐fibres CV (m/s ± SEM)	*n* = 22 16.10 ± 0.57	*n* = 27 12.31 ± 0.37	*n* = 12 10.48 ± 0.45	*F*(2, 58) = 30.79 *P* < 0.0001
SAMs CV (m/s)	*n* = 11 15.27 ± 0.67	*n* = 14 12.71 ± 0.58	*n* = 8 10.48 ± 0.53	*F*(2, 30) = 12.75 *P* < 0.0001
RAMs CV (m/s)	*n* = 11 16.93 ± 0.89	*n* = 13 11.87 ± 0.45	*n* = 4 10.48 ± 0.94	*F*(2, 25) = 19.23 *P* < 0.0001
Aδ‐fibres CV (m/s)	*n* = 14 5.91 ± 0.37	*n* = 16 5.13 ± 0.3904	*n* = 6 4.19 ± 0.61	*F*(2, 33) = 2.957 *P* = 0.0659
AMs CV (m/s)	*n* = 8 6.16 ± 0.62	*n* = 11 4.74 ± 0.51	*n* = 6 4.19 ± 0.61	*F*(2, 22) = 2.738 *P* = 0.0868
D‐hairs CV (m/s)	*n* = 6 5.58 ± 0.2765	*n* = 5 5.98 ± 0.34	*n* = 0	*P* = 0.3771

*Note*: The mean values ± SEM are listed. Datasets were compared using one‐way ANOVAs or Student's two‐tailed unpaired *t*‐test.

Abbreviations: AM, A‐mechanonociceptor; CV, conduction velocity; D‐hair, down‐hair receptor; RAM, rapidly adapting mechanoreceptor, SAM, slowly adapting mechanoreceptor.

We next assessed the stimulus–response functions of muscle afferents innervating the skin compared with those of regenerated and intact cutaneous afferents. Normal RAMs are tuned primarily to stimulus velocity (Walcher et al., 2018), and therefore we used a series of stimuli of increasing velocity (ramp‐and‐hold stimuli with probe velocities of 0.075, 0.15, 0.45 and 1.5 mm/s) at a constant displacement of 96 µm to probe mechanoreceptor sensitivity. In addition, we used a sinusoidal vibration stimulus (50 Hz) applied with increasing amplitude to determine the minimal mechanical threshold to activate mechanoreceptors. No significant differences in the stimulus–response properties of RAMs were observed across the three experimental groups (example traces are shown in Figure 2c); spike frequencies of RAMs in response to moving stimuli were similar across the groups. The response properties of SAMs were examined using a series of stimuli of increasing displacement with a constant ramp velocity. The displacements ranged from 15 to 250 mN and lasted for 2 s during the hold phase. Spike frequencies (in herz) were calculated by counting the number of spikes occurring during the hold phase of the stimulus. Mechanical thresholds (in millinewtons) were assessed by measuring the minimal force needed to evoke an action potential, that is, by measuring the force at which the first action potential occurred during the dynamic phase of the stimulus. In all three preparations (‘intact’, ‘self’ and ‘cross’), characteristic SAM responses were recorded (example traces are shown in Figure 2f). However, the stimulus–response functions of SAMs in cross‐anastomosed gastrocnemius nerve preparations were significantly larger in the suprathreshold range compared with intact and self‐anastomosed sural nerve preparations. Super‐sensitive SAM responses in the cross‐anastomosed nerve might reflect the intrinsic properties of former muscle spindle afferents that can sustain extremely high firing rates. No significant differences in the mechanical thresholds of SAMs were observed between the three preparations.

No sensory fibres with physiological attributes of D‐hair receptors were found in the cross‐anastomosed gastrocnemius nerve. However, the stimulus–response properties of D‐hair receptors found in intact and self‐anastomosed sural nerves were not different (example traces are shown in Figure 2i). Both spike frequencies and mechanical thresholds were essentially identical between intact and self‐anastomosed sural nerves (Figure S1a,b), indicating that D‐hair receptors easily regain their functional properties following nerve lesion. Characteristic AM responses were found in all three preparations (examples traces are shown in Figure 2j). The response properties of AMs, including spike frequencies and mechanical thresholds, were not significantly different from each other (Figure S1c,d).

### 3.2 STOML3 is required for muscle afferents to acquire mechanosensitivity in the skin

**FIGURE 3:**
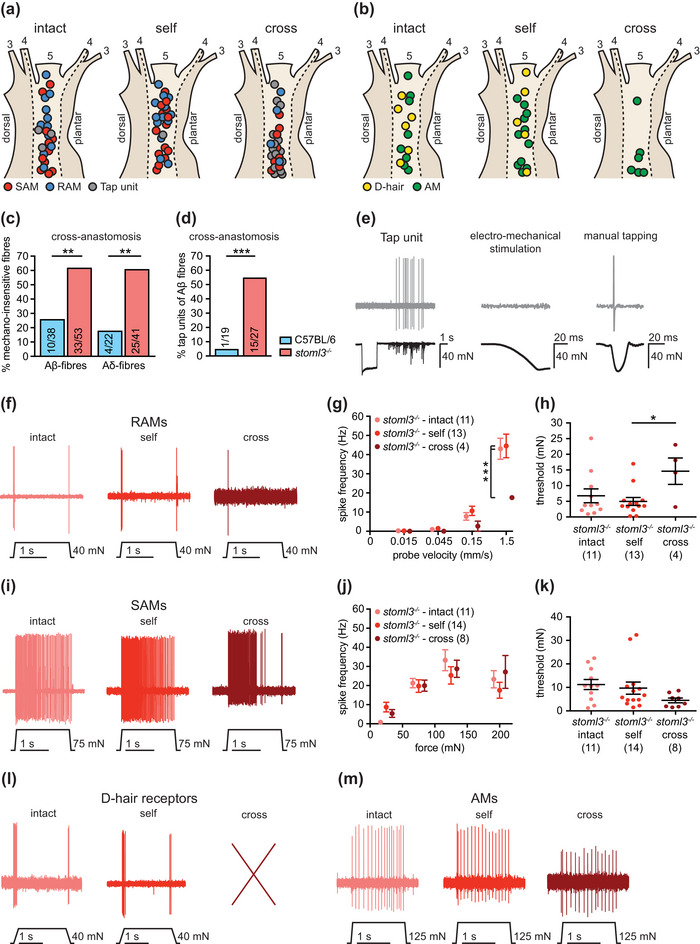
Response properties of muscle afferents newly innervating the skin in comparison to intact and regenerated cutaneous afferents in *stoml3* mutant mice. (a, b) Receptive field locations of intact cutaneous, regenerated cutaneous and redirected muscle afferent Aβ‐fibres (a; SAMs, red; RAMs, blue; tap‐units, grey) and Aδ‐fibres (b; D‐hair receptors, yellow; AMs, green). (c) Proportions of mechano‐insensitive Aβ‐ and Aδ‐fibres in the skin territory of the sural nerve innervated by redirected muscle afferents in *stoml3* mutant (red, *n* = 3) in comparison to control (blue, *n* = 4) mice was significantly different; two‐sided Fisher's exact test: *P* = 0.001 for both Aβ‐ and Aδ‐fibres. (d) Proportion of units exhibiting tap‐unit responses in skin innervated by redirected muscle afferents in *stoml3* mutant (red, *n* = 10) compared with control (blue, *n* = 9) mice. These differences were statistically significant; two‐sided Fisher's exact test: *P* < 0.0001. (e) Example trace of a tap‐unit only responding to manually delivered brisk tapping, but not to a controlled stimulus delivered by mechanoelectrical stimulator. (f) Example traces of RAM responses to a ramp‐and‐hold stimulus with a probe velocity of 1.5 mm/s. (g) Spike frequencies of RAMs in response to ramp‐and‐hold stimuli with increasing ramp velocities. Mean values ± SEM are shown. Statistical differences were calculated with a two‐way repeated‐measures ANOVA: *F*(6, 75) = 3.136, *P* = 0.009; Bonferroni's *post hoc* test. (h) Mechanical thresholds of RAMs measured in response to a sinusoidal vibration stimulus (50 Hz). Individual data points and mean values ± SEM are shown. Statistical significance was calculated with a one‐way ANOVA: *F*(2, 25) = 3.587, *P* = 0.043; Tukey's multiple comparison test. (i) Example traces of SAM responses to a ramp‐and‐hold stimulus, 75 mN indentation force. (j) Spike frequencies of SAMs in response to a series of increasing displacement stimuli were not different between groups; two‐way repeated‐measures ANOVA: *F*(6, 90) = 1.532, *P* = 0.177. Mean values ± SEM are shown. (k) Mechanical thresholds for SAMs were also not statistically different between groups; one‐way ANOVA: *F*(2, 28) = 1.466, *P* = 0.2479. Individual data points and mean values ± SEM are shown. (l) Example traces of D‐hair receptor responses to a ramp‐and‐hold stimulus with a probe velocity of 0.45 mm/s. (m) Example traces of AM responses to a ramp‐and‐hold stimulus, 125 mN indentation force. Abbreviations: AM, A‐mechanonociceptor; D‐hair, Down‐hair; RAM, rapidly adapting mechanoreceptor; SAM, slowly adapting mechanoreceptor.

In *stoml3* mutant mice, mechanosensitive Aβ‐fibres, including SAMs and RAMs (Figure 3a), and AM fibres (Figure 3b), were found in all three experimental groups (‘intact’, ‘self’ and ‘cross’). As in wild‐type mice, D‐hair receptors were recorded only in the intact and self‐anastomosed sural nerves (Figure 3b). In addition, we found tap‐units in all three preparations. These were afferents that fired only one spike in response to extremely rapid, high‐amplitude mechanical stimulation (Figure 3a). Such units were commonly encountered in *stoml3* mutant mice in our previous studies (Wetzel et al., 2007). It was immediately obvious that it was very difficult to find mechanosensitive afferent fibres in the cross‐anastomosed gastrocnemius nerve. Indeed, most fibres with a receptive field were found to be so‐called tap‐units (Figure 3a–e). Considering the sparsity of responsive fibres found in the cross‐anastomosed nerve in *stoml3* mutant mice, we adopted an electrical search protocol (Chakrabarti et al., 2024; Wetzel et al., 2007) to assess the proportion of fibres with apparently no mechanosensitive receptive field in the cross‐anastomosed gastrocnemius nerve. We found a dramatic and statistically significant increase in the proportion of mechano‐insensitive Aβ‐fibres and Aδ‐fibres in the cross‐anastomosed gastrocnemius nerve innervating the skin in *stoml3* mutants compared with controls [Aδ‐fibres: C57BL/6, 26% (4 of 22 fibres), *n* = 4 mice; *Stoml3* ^−/−^, 62% (25 of 41 fibres), *n* = 3 mice; Aβ‐fibres: C57BL/6, 18% (10 of 38 fibres), *n* = 4 mice; *stoml3* ^−/−^, 61% (33 of 53 fibres), *n* = 3 mice; Figure 3c]. Of the remaining mechanosensitive Aβ‐fibres in *stoml3* mutant mice (∼39% of the total fibres), more than half (55%) were classified as tap‐units in the cross‐anastomosed nerve. This was in marked contrast to wild‐type cross‐anastomosed nerves, in which only 5% of the already large number of mechanosensitive fibres (82% of all fibres) were classified as tap‐units. The large increase in the number of tap‐units was statistically different between wild‐type and *stoml3* mutants [C57BL/6, 5% (1 of 19 fibres), *n* = 9 mice; *stoml3* ^−/−^, 55% (15 of27 fibres), *n* = 10 mice; example traces are shown in Figure 3d, e]. Tap‐units clearly represent sensory fibres that would be extremely difficult to activate by natural touch stimuli in vivo.

We next assessed the stimulus–response properties of the remaining mechanosensitive Aβ‐mechanoreceptors in the cross‐anastomosed gastrocnemius nerve (Table 1; example traces are shown in Figure 3f, i), which we estimated to be ∼18% of all fibres in *stoml3* mutants compared with 78% in controls. The suprathreshold responses to moving stimuli of RAMs found in the cross‐anastomosed gastrocnemius nerve preparation were significantly decreased compared with RAMs found in the intact or self‐anastomosed nerve, and this was particularly prominent for the fastest stimulus of 1.5 mm/s (‘intact’, *n* = 11 fibres; ‘self’, *n* = 13 fibres; ‘cross’, *n* = 4 fibres; Figure 3g). In addition, mechanical thresholds of RAMs were found to be significantly higher in cross‐anastomosed muscle nerves compared with self‐anastomosed nerves (‘intact’, 6.8 ± 2.2 mN, *n* = 11 fibres; ‘self’, 5.0 ± 1.3 mN, *n* = 13 fibres; ‘cross’, 14.6 ± 4.2 mN, *n* = 4 fibres; Figure 3h). Interestingly, the few sensory afferents found with properties of SAMs in the cross‐anastomosed gastrocnemius nerve were indistinguishable from those in intact or self‐anastomosed nerves (stimulus–response function shown in Figure 3j; mechanical thresholds shown in Figure 3k).

Unlike the findings in wild‐type mice, D‐hair receptors found in the self‐anastomosed *stoml3* mutant sural nerve fired significantly fewer spikes in response to ramp‐and‐hold stimuli compared with D‐hair receptors in the intact *stoml3* mutant sural nerve (‘intact’, *n* = 6 fibres; ‘self’, *n* = 5 fibres; examples traces are shown in Figure 3l, Figure S2a). However, there was no difference in mechanical thresholds between D‐hair receptors found in *stoml3* mutant mice within the intact or self‐anastomosed nerve (‘intact’, 0.7 ± 0.4 mN, *n* = 6 fibres; ‘self’, 0.6 ± 0.2 mN, *n* = 5 fibres; Student's two‐tailed unpaired *t*‐test: *t*(9) = 0.1511, *P* = 0.883; Figure 3l Figure S2b). Thinly myelinated nociceptors or AMs recorded from *stoml3* mutants (example traces are shown in Figure 3m) exhibited similar stimulus–response functions and mechanical thresholds in intact, self‐ and cross‐anastomosed nerves (Figure S2c,d).

**FIGURE 4:**
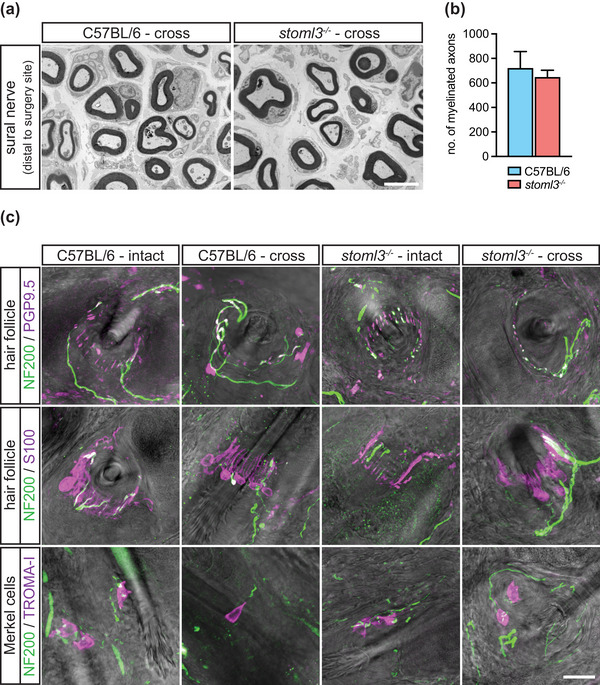
Skin innervation by muscle afferents in *stoml3* mutant and control mice. (a) Transverse electron microscopic images of cross‐anastomosed gastrocnemius nerves innervating the skin distal to the surgery site in *stoml3* mutant and wild‐type mice. Scale bar: 3 µm. (b) Numbers of myelinated fibres counted in cross‐anastomosed sural nerves distal to the surgery site in *stoml3* mutant (red, *n* = 3) in comparison to control (*n* = 3, blue) mice; no significant difference, Student's two‐tailed unpaired *t*‐test: *t*(4) = 0.5061, *P* = 0.6394. Mean values ± SEM are shown. (c) Fluorescence images of hair follicles and Merkel cells in the skin territory of the sural nerve innervated by intact sural nerve afferents and redirected muscle afferents. Scale bar: 20 µm. Abbreviations: NF200, neurofilament 200; PGP9.5, protein gene product 9.5; TROMA‐I, trophectodermal monoclonal antibody against cytokeratin 8.

The striking lack of mechanosensitive fibres in the cross‐anastomosed gastrocnemius nerve might have been attributable to an inability of sensory fibres to regenerate and form appropriate endings in *stoml3* mutant mice. We used transmission electron microscopy to quantify the number of myelinated axons that regenerated distal to the cross‐anastomosis site in wild‐type and *stoml3* mutant mice (*n* = 3 mice each; representative images are shown in Figure 4a). We found equal numbers of regenerated axons in both genotypes (C57BL/6 ‘cross’, 722.4 ± 133.9, *n* = 3 mice; *stoml3* ^−/−^ ‘cross’, 649.2 ± 54.92, *n* = 3 mice; Figure 4b). We also assessed the innervation of the skin by redirected muscle afferents in both controls and *stoml3* mutants. Using immunocytochemistry to label all sensory fibres with antibodies against protein gene product 9.5 (PGP9.5) or myelinated sensory fibres with antibodies against neurofilament 200 (NF200), we could show that hair follicles were innervated by muscle sensory afferents (*n* = 3 mice each; representative images are shown in Figure 4c). Thus, lanceolate endings positive and negative for NF200 were found in the skin innervated by the muscle nerve in both wild‐type and *stoml3* mutant mice. Furthermore, these endings were similar to those found in the intact sural nerve territory. We also labelled the endings of putative SAMs in the skin using antibodies against cytokeratin 8/18 (TROMA‐I) to label Merkel cells and found that these cells were innervated by muscle sensory axons positive for NF200 in both wild‐type and *stoml3* mutant mice (*n* = 3 mice each; representative images are shown in Figure 4c). We conclude that the remarkable ability of muscle afferents to form sensory endings appropriate for the skin does not, in fact, depend on the presence of STOML3.

### 3.3 Somatotopic map formation is blurred in *stoml3* mutant mice

Previous studies in rats have shown a remarkable amount of functional plasticity of muscle afferents redirected to the skin. Redirected muscle afferents engage new reflexes and make new synaptic connections with dorsal horn neurons in a somatotopically appropriate manner (Lewin & McMahon, 1993; McMahon & Wall, 1989). In order to study the structural plasticity of sensory afferents after regeneration, we established a quantitative method to study somatotopic mapping of sensory afferent terminals in the spinal cord (Tröster et al., 2018). We first used this tracing methodology to map the accuracy of sensory afferent projections in touch‐deficient *stoml3* mutant mice, because the presence of deficits in the intact condition could have a bearing on what happens after nerve regeneration. Sensory afferents innervating the second and third digit of the left and right hind paw, respectively, were labelled using subcutaneous injections of cholera toxin subunit B conjugated with Alexa Fluor 594 (CTB), which is endocytosed selectively by myelinated fibres (Robertson & Arvidsson, 1985; Wan et al., 1982). Five days after the injection, the central terminal fields of cutaneous fibres innervating the skin of the second and third digit of the left and right hind paw, respectively, were mapped in their entirety in unsectioned cleared spinal cords of 4‐week‐old *stoml3* mutant and control mice. Control mice were the C57BL/6 strain, because the *stoml3* mutant line had been backcrossed onto the same background for ≥10 generations. To visualize CTB‐labelled projections, we evaluated several of the published optical clearing methods (Costantini et al., 2015; Ertürk et al., 2011, 2012; Kloepper et al., 2010; Staudt et al., 2007) and found that immersion clearing using 2′2‐thiodiethanol (TDE) produced the least tissue shrinkage whilst providing sufficient imaging depth and preserving fluorescence intensity (Figure S3).

**FIGURE 5:**
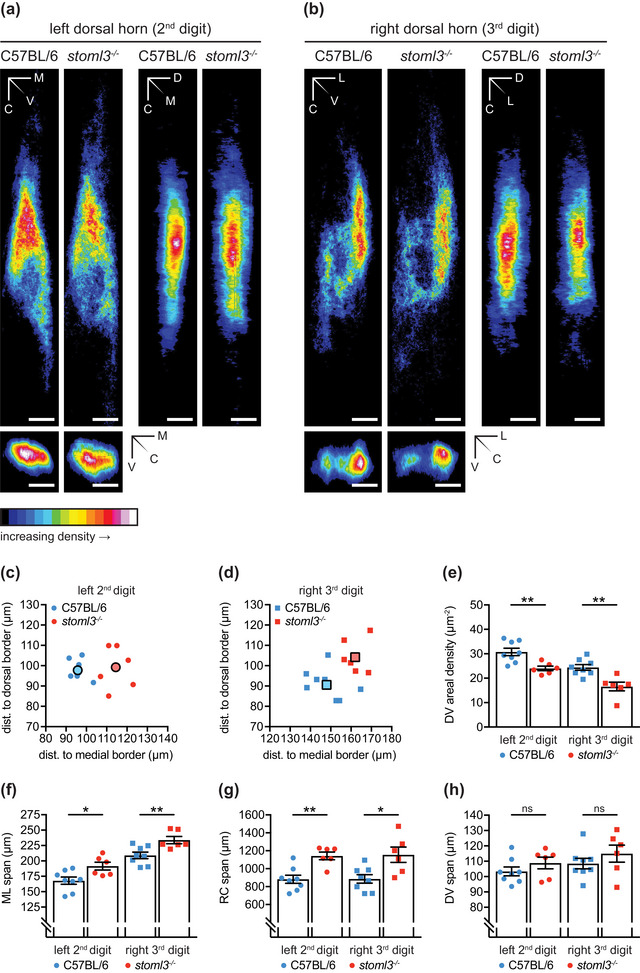
Morphometric and density measurements of spinal terminal fields of fibres innervating the left second and right third hind paw digit in *stoml3* mutant and control mice. (a, b) Averaged summed dorsoventral, mediolateral and rostrocaudal projections of spinal terminal fields of fibres innervating the left second (a) and right third (b) hind paw digit in *stoml3* mutant (*n* = 6) and control (*n* = 8) mice. The ImageJ colour look‐up table ‘16 Colors’ was applied. The colour code in each pixel denotes the number of voxels found in corresponding positions along different axes averaged across mice. Scale bars: 100 µm. (c, d) The locations of terminal field foci of fibres innervating the second digit of the left hind paw (circles; c) and the third digit of the right hind paw (squares; d) relative to the medial and dorsal grey–white matter border in *stoml3* mutant (red, *n* = 6) and control (blue, *n* = 8) mice. Individual data points are shown. (e) The dorsoventral density was significantly lower in *stoml3* mutant mice for the left terminal field: C57BL/6, 30.8 ± 1.5 voxels/µm^2^, *n* = 8 mice; *stoml3* ^−/−^, 24.0 ± 0.9 voxels/µm^2^, *n* = 6 mice; Student's two‐tailed unpaired *t*‐test: *t*(12) = 3.463, *P* = 0.005; and the right terminal field: C57BL/6, 24.4 ± 1.2 voxels/µm^2^, *n* = 8 mice; *stoml3* ^−/−^, 16.5 ± 1.8 voxels/µm^2^, *n* = 6 mice; Student's two‐tailed unpaired *t*‐test: *t*(12) = 3.848, *P* = 0.002. (f–h) Spans of terminal fields of fibres innervating the left second (circles) and right third (squares) hind paw digit in the mediolateral (ML; f), rostrocaudal (RC; g) and dorsoventral (DV; h) dimension in *stoml3* mutant (red, *n* = 6) and control (blue, *n* = 8) mice. Individual data points and mean values ± SEM are shown. Each dataset was compared using Student's two‐tailed unpaired *t*‐test. (e) Areal densities of terminal fields innervating the left second (circles) and right third (squares) hind paw digit in dorsoventral projections in *stoml3* mutant (red, *n* = 6) and control (blue, *n* = 8) mice. Individual data points and mean values ± SEM are shown. Each dataset was compared using a two‐tailed unpaired *t*‐test. Abbreviations: C, caudal; D, dorsal; DV, dorsoventral; L, lateral; M, medial; ML, mediolateral; RC, rostrocaudal; V, ventral.

We performed experiments to ensure that a comparative analysis of CTB‐labelled spinal terminal fields could be made between *stoml3* mutants and controls. Importantly, the mediolateral widths of spinal cord dorsal horns, measured at a depth of 80 µm from the dorsal surface, were not different in *stoml3* mutants compared with controls (C57BL/6, 1091 ± 20 µm, *n* = 8 mice; *stoml3* ^−/−^, 1114 ± 16 µm, *n* = 6 mice; Figure 5; Figure S4a). Furthermore, reliable and consistent numbers of sensory neurons were labelled, as demonstrated by counting total numbers of CTB‐labelled neurons in lumbar DRGs 3, 4 and 5, which innervate the hindlimb skin; on average, there was no difference in the numbers of labelled sensory neurons between genotypes (second left hind paw digit: C57BL6, 124.2 ± 7.8, *n* = 5 mice; *stoml3* ^−/−^, 127.6 ± 8.6, *n* = 5 mice; third right hind paw digit: C57BL6, 132.6 ± 13.2, *n* = 5 mice; *stoml3* ^−/−^, 129.6 ± 3.9, *n* = 5 mice; Figure S4b–2b). Furthermore, CTB labelling was restricted to the same skin areas in both control and *stoml3* mutant mice (*n* = 3 mice each; representative images are shown in Figure S4c).

The segmental and laminar location of CTB‐labelled terminals and their overall geometry were similar between genotypes (Figure 5a, b). For quantitative analyses, the three‐dimensional centres of mass of the voxel clouds representing CTB‐labelled fibre terminals in binary image stacks were measured, and the terminal fields were aligned to their centre of mass (Figure 5; Figure S5; Video S1). Summed dorsoventral, rostrocaudal and/or mediolateral projections of the binary image stacks were constructed, and a colour look‐up table was applied to enable visualization of terminal fields in *stoml3* mutant and control mice (Figure 5a, b; Video S1). First, we determined the locations of the terminal field foci relative to the medial and dorsal grey–white matter border in *stoml3* mutant and control mice. The foci of terminal fields of fibres innervating the second digit were, on average, shifted laterally by 18.75 µm and ventrally by 1.42 µm in *stoml3* mutant mice compared with controls (Figure 5c), and the foci of labelled terminal fields representing the third digit were, on average, shifted laterally by 14.00 µm and ventrally by 13.54 µm in *stoml3* mutant mice compared with controls (Figure 5d). In the two‐dimensional space, the foci of the terminal fields of fibres innervating the second and third digit were, on average, shifted by 18.8 µm and 19.5 µm, respectively, in *stoml3* mutant mice compared with controls (Figure 5c, d). The terminal field foci tended to be shifted in a rostrolateral direction, as can be seen by examining the summed termination fields (Figure 5a, b). Next, we determined the maximal extent of the terminal fields in the mediolateral, rostrocaudal and dorsoventral dimensions by measuring the dimensions of the minimum bounding rectangle that enclosed all pixels in dorsoventral and rostrocaudal summed projections of the binary image stacks. The extents of the terminal fields in *stoml3* mutant and control mice were found to be significantly different (Table 2; Figure 5f–h). The spinal terminal fields of fibres innervating the second and third digit of the left and right hind paw, respectively, extended 14% and 12% further in the mediolateral dimension (Table 2; Figure 2f) and 30% in the rostrocaudal dimension in *stoml3* mutant mice compared with control mice (Table 1; Figure 2g). No differences in the extent of terminal fields in the dorsoventral dimension were observed between genotypes (Table 2; Figure 2h). Despite the fact that terminal fields were expanded in *stoml3* mutants, the numbers of voxels representing CTB labelling was not different between the two genotypes (left terminal field: C57BL/6, 3.14 ± 0.418 × 10^6^, *n* = 8 mice; *stoml3* ^−/−^, 2.78 ± 0.183 × 10^6^, *n* = 6 mice; right terminal field: C57BL/6, 2.30 ± 0.271 × 10^6^, *n* = 8 mice; *stoml3* ^−/−^, 1.79 ± 0.260 × 10^6^, *n* = 6 mice; Figure 5; Figure S5). The lack of change in voxel numbers led us to suspect that there was a decrease in the density of the spinal terminal fields in *stoml3* mutants compared with controls, which was already apparent in the summed intensity projections shown in Figure 5a, b. We calculated the areal density of the terminal fields separately in the dorsoventral, mediolateral and rostrocaudal summed projections by dividing the total number of voxels in binary image stacks by the area occupied by pixels in each of the three projections (Figure 5; Figure S5). The areal densities of terminal fields of fibres innervating the second and third digits, respectively, were significantly lower in all three projections in *stoml3* mutants compared with control mice (Figure 5e; Figure S5b–d). In dorsoventral projections, the areal density of fibres innervating the hind paw digits were between 22% and 32% less dense in *stoml3* mutants compared with controls, and this was statistically significant (Figure 5e). Our quantitative analysis demonstrates that afferent terminal fields in the deep dorsal horn occupy a larger area of the spinal cord and are less dense in *stoml3* mutant mice compared with controls.

**TABLE 2:** Morphometric measurements of spinal terminal fields in *stoml3* mutant in comparison to control mice.

	C57BL/6 (*n* = 8)	*stoml3^−^* ^/^ *^−^* (*n* = 6)	Statistics
Left dorsal horn			
ML span (µm)	167.9 ± 5.9	191.6 ± 6.3	*t*(12) = 2.720, *P* = 0.01
RC span (µm)	882.0 ± 45.6	1143.0 ± 42.3	*t*(12) = 4.051, *P* = 0.002
DV span (µm)	103.4 ± 2.9	108.9 ± 3.8	*t*(12) = 1.170, *P* = 0.265
**Right dorsal horn**			
ML span (µm)	209.0 ± 5.3	233.9 ± 5.8	*t*(12) = 3.154, *P* = 0.008
RC span (µm)	885.7 ± 47.0	1155.0 ± 85.4	*t*(12) = 2.950, *P* = 0.012
DV span (µm)	108.5 ± 3.4	114.9 ± 5.6	*t*(12) = 1.034, *P* = 0.321

*Note*: The mean values ± SEM are listed. Datasets were compared using one‐way ANOVAs or Student's two‐tailed unpaired *t*‐test.

Abbreviations: DV, dorsoventral; ML, mediolateral; RC, rostrocaudal.

### 3.4 Mechanosensory silence does not prevent structural plasticity

**FIGURE 6:**
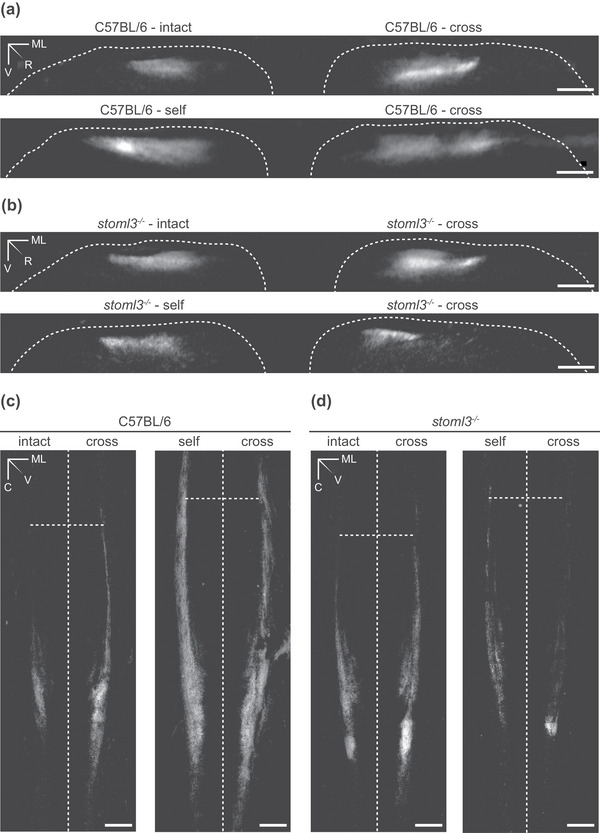
Spinal terminal fields of intact, self‐ and cross‐anastomosed nerve afferents innervating the sural nerve skin territory in wild‐type and *stoml3* mutant mice. (a, b) Examples of rostrocaudal projections of terminal fields of muscle afferents redirected towards the skin in comparison to intact and regenerated cutaneous afferents in wild‐type (a) and *stoml3* mutant (b) mice. The dorsal grey–white matter border is marked with a white dashed line. Scale bars: 100 µm. (c, d) Summed dorsoventral projections of terminal fields innervating of muscle afferents redirected towards the skin in comparison to intact and regenerated cutaneous afferents in wild‐type mice (c) and *stoml3* mutant mice (d). Vertical dashed lines mark the posterior median sulcus, and horizontal dashed lines mark the border between spinal lumbar segments 3 and 4. Scale bars: 200 µm. Abbreviations: C, caudal; ML, mediolateral; R, rostral; V, ventral.

We next asked whether we could use CTB labelling to visualize the structural plasticity of muscle afferents in the dorsal horn. Normally myelinated sensory fibres from the gastrocnemius muscle (proprioceptors and myelinated muscle nociceptors) terminate in deeper dorsal laminae and predominantly in the ventral horn (Brown, 1981; Brown & Fyffe, 1978, 1979), whereas skin mechanoreceptors project to (inter‐)neurons in laminae III–V in a somatotopically organized fashion (Shortland & Woolf, 1993; Shortland et al., 1989; Wetzel et al., 2007). We labelled axons within the regenerated nerve by injecting 2 µL CTB into the nerve distal to the anastomosis site and waited 5 days before visualizing the transganglionically transported tracer in the dorsal horn (Wetzel et al., 2007). We could reliably trace the central terminals of intact and regenerated sural nerve fibres and cross‐anastomosed muscle afferents newly innervating the skin in both wild‐type and *stoml3* mutant mice (Figure 6). The tributary branches of the sciatic nerve, namely the cross‐anastomosed gastrocnemius and sural nerves, the common peroneal nerve and the tibial nerve, were examined microscopically for CTB labelling to ensure restricted labelling of fibres running in the cross‐anastomosed gastrocnemius nerve. Only the sural nerve trunk containing redirected gastrocnemius sensory fibres was labelled (*n* = 3 mice each; representative images are shown in Figure 6; Figure S6).

The raw images of CTB‐labelled terminals in the spinal cord were binarized, and summed dorsoventral and rostrocaudal projections were generated to assess the laminar positioning and the somatotopic arrangement of the terminal fields. As shown in Figure 6a (representative images are shown), the central terminals of muscle afferents newly innervating the skin terminated at the same dorsoventral level as cutaneous afferents of the intact and self‐anastomosed sural nerve (‘intact’ and ‘self’, *n* = 3 mice each; ‘cross’, *n* = 7 mice; representative images are shown in Figure 6a). Furthermore, the central terminals of muscle afferents established somatotopically organized projections resembling the terminal fields of intact and self‐anastomosed sural nerves (‘intact’ and ‘self’, *n* = 3 mice each; ‘cross’, *n* = 7 mice; representative images are shown in Figure 6c). Injections of CTB into the popliteal fossa of mice in which the sural nerve was transected did not lead to labelling of central afferents. Surprisingly, spinal terminals of muscle afferents now innervating the skin in *stoml3* mutant mice reorganized to terminate in a somatotopically organized fashion in dorsal horn laminae comparable to those of the intact and regenerated sural nerve (‘intact’ and ‘self’, *n* = 3 mice each; ‘cross’, *n* = 6 mice; representative images are shown in Figure 6b, d). Thus, we have demonstrated that muscle afferents confronted with a new target in the skin can exhibit substantial structural plasticity in that they form new anatomical connections with somatotopically appropriate dorsal horn neurons. Strikingly, a substantial loss of mechanosensitivity in most of the redirected gastrocnemius afferents in the skin of *stoml3* mutant mice does not prevent these afferents from displaying similar structural plasticity to controls.

## 4 DISCUSSION

In the rat and cat, it was shown that muscle and cutaneous afferents redirected towards inappropriate targets (i.e., muscle afferents to skin and vice versa) gain neurochemical and physiological properties appropriate for their new target (McMahon & Gibson, 1987; McMahon & Wall, 1989; Johnson et al., 1995; Lewin & McMahon, 1991a, 1991b). It has been known for some time that regenerating axons efficiently regain receptor properties in their new target after nerve transection (Burgess & Horch, 1973; Fawcett & Keynes, 1990). However, to date nothing was known about the molecular factors that are required for the re‐acquisition of mechanosensitive receptive fields after regeneration. In contrast to wild‐type mice, in which virtually all muscle afferents can make mechanosensitive endings in the skin (Figure 2), when directed to the skin only a small fraction (<20%) of muscle afferents from *stoml3* mutants were capable of acquiring normal mechanosensitivity (Figure 3). Only muscle afferents that acquired the properties of SAMs showed mechanosensitivity similar to controls (Figure 3). We presume that these muscle afferents form peripheral endings associated with Merkel cells (Figure 4), which themselves contribute to SAM mechanosensitivity (Maksimovic et al., 2014; Ojeda‐Alonso et al., 2024). Thus, *stoml3* appears to be required genetically for most muscle afferents to form mechanosensitive endings in the skin. Despite the lack of mechanosensory function, muscle afferents formed morphological end‐organs in the skin appropriate to the new target in the absence of *stoml3* (Figure 4). Other molecular factors have recently been identified (e.g., *Meis2*) that are required for the anatomical formation of sensory endings during development (Desiderio et al., 2024). In contrast, the STOML3 protein is dispensable for the formation of end‐organ morphology, but is still required for most muscle afferents to acquire mechanosensitivity in the skin.

Following re‐routing to skin, muscle afferents display remarkable functional plasticity in the spinal cord (Lewin & McMahon, 1993; McMahon & Wall, 1989), forming somatotopically appropriate connections to dorsal horn neurons that normally receive little synaptic input from intact muscle afferents (Lewin & McMahon, 1993). Here, we show that there is a substantial anatomical rearrangement of the central terminals of myelinated muscle afferents after re‐routing to skin. The synaptic terminals of muscle afferents innervating the skin could be visualized robustly after CTB tracing in a restricted region of the dorsal horn that corresponds to the appropriate somatotopic territory occupied by afferents from the intact or self‐anastomosed sural nerve (Figure 6). This anatomical plasticity was robust and was observed in all animals studied, including *Stoml3* mutant mice. Muscle afferents do not normally project to the same region of the dorsal horn before regeneration, but this was difficult to show directly. We carried out CTB‐tracing experiments from the intact gastrocnemius nerve but never observed any signal in the cleared spinal cord after two‐photon imaging. It was impossible to tell in these circumstances whether the tracing had failed (a rare occurrence in our hands) or whether, as previously documented, the muscle afferent synapses in the dorsal horn are so sparse (Hoheisel et al., 1989; Molander & Grant, 1987; Panneton et al., 2005) that labelling was not detectable in the cleared tissue. This striking structural plasticity also occurred in *stoml3* mutant mice despite an almost complete loss of mechanosensitivity of muscle afferents innervating the skin (Figure 3). We could not detect any major difference in the central projection of afferents from muscle nerves innervating skin between wild‐type and *stoml3* mutant mice. However, the variability in the central projections of cross‐anastomosed muscle afferents between animals made it impossible to quantify differences in projection patterns between genotypes reliably. If activity arising from the periphery plays a role, it could be that the reduced mechanically evoked activity in *stoml3* mutant mice is still sufficient over time to direct anatomical plasticity.

Using a precise and unbiased method to reconstruct the somatotopy of cutaneous projections after CTB tracing, we observed a more diffuse and dispersed representation of the skin in the spinal cord of *stoml3* mutant mice (Figure 5). Around 40% of mechanoreceptors in *stoml3* mutant mice are mechanically silent (Wetzel et al., 2007, 2017), and we speculate that the lack of stimulus‐evoked activity might have impaired activity‐dependent sharpening of the somatotopic map in these animals (Beggs et al., 2002; Granmo et al., 2008). Indeed, the diffuse somatotopic map that we observed here might be a major reason for reduced tactile acuity in *stoml3* mutant mice (Wetzel et al., 2007). In these experiments, we found that each digit is represented within a rostrocaudal band, which, at its narrowest, has a width of <100 µm (Figure 5). Considering the substantial expansion (≤30%) of the terminal fields in *stoml3* mutants, it is clear that the representation of the digits will overlap. Unfortunately, we could not visualize such an overlap directly, because the clearing methodology was not compatible with using two different fluorescent CTB conjugates.

It is well established that after nerve transection, sensory axons reach topologically inappropriate positions in the skin and may not reinnervate the same end‐organ as before the lesion (Burgess & Horch, 1973; Johnson et al., 1995; Lewin et al., 1994). An extreme case of adult plasticity is when muscle afferents are forced to regenerate inappropriately to skin, a situation that undoubtedly happens following mixed nerve injury in humans (Rbia & Shin, 2017). The only skin receptor type not found in the cross‐anastomosed gastrocnemius nerve in both genotypes was D‐hair receptors (Figure 2). The D‐hair receptors are the most sensitive type of skin mechanoreceptor. They have thinly myelinated axons and are characterized by high expression of the T‐type calcium channel Ca_V_3.2 (Bernal Sierra et al., 2017; Lechner & Lewin, 2013; Shin et al., 2003; Walcher et al., 2018; Wang & Lewin, 2011). There is no evidence that the normal muscle is innervated by thinly myelinated low‐threshold mechanoreceptors (Mense, 1996), which suggests that nociceptors from the muscle can only acquire properties of nociceptors in the skin.

Mutant mice lacking the PIEZO2 modulating protein STOML3 have deficits in tactile acuity (Wetzel et al., 2007), but unlike PIEZO2‐deficient humans and mice, do not have proprioceptive deficits (Chesler et al., 2016; Ranade et al., 2014; Wetzel et al., 2007; Woo et al., 2015). Thus, STOML3 might not be expressed normally in muscle proprioceptors. Here, we find that the presence of STOML3 is required for the vast majority of muscle afferents to form mechanosensitive receptive fields in the skin (Figure 3). These data are consistent with the hypothesis that *de novo* expression of STOML3 in muscle afferents innervating the skin is a prerequisite for mechanosensitivity. Indeed, we have shown that nerve injury alone is sufficient to upregulate STOML3 protein in sensory neurons (Wetzel et al., 2017), but it is also possible that signals in the skin instruct muscle afferents to express *stoml3*. One example of a peptide factor that has high expression in skin but low expression in muscle and can drive spinal plasticity is nerve growth factor (NGF) (Korsching & Thoenen, 1983; Lewin et al., 1992; Shelton & Reichardt, 1984). However, NGF does not upregulate STOML3 expression in the DRG (Wetzel et al., 2017), but could play a role in driving central plasticity (Lewin et al., 1992). The effects of *stoml3* loss of function were highly specific, because muscle afferents were capable of regenerating to the skin and forming morphologically appropriate sensory endings in the skin without *stoml3*. Regeneration was robust in all cases, because similar numbers of myelinated gastrocnemius fibres were present in the distal sural nerve stump after cross‐anastomosis in wild‐type and *stoml3* mutant mice (Figure 4).

Recent work in flies has established a link between mechanotransduction and regeneration (Song et al., 2019). Here, we examined the role of the mechanotransduction protein STOML3 in peripheral nerve regeneration. Sensory axons were able to regenerate and form specialized end‐organ morphologies in the absence of the STOML3 protein (Figure 4). However, we found that the presence of STOML3 was necessary for muscle afferents to acquire normal mechanosensitivity in the skin. We also found that the somatotopic organization of cutaneous afferents in the dorsal horn is correct, but significantly less focused and precise in *stoml3* mutants compared with controls (Figure 5). Nevertheless, the central terminals of muscle afferents in the *stoml3* mutant mice exhibit dramatic structural plasticity, forming somatotopically appropriate terminals even when stimulus‐evoked activity was greatly attenuated compared with controls. We conclude that there are likely to be chemical factors in the skin that can induce expression of STOML3 in muscle afferents and direct sprouting of their central terminals into somatotopically appropriate areas of the spinal dorsal horn. However, sensory‐evoked activity, even at a low level, might still contribute to this plasticity.

## AUTHOR CONTRIBUTIONS

Julia Haseleu carried out all experimental work, including imaging and electrophysiology. Jan Walcher helped with electrophysiology experiments and analysis. Gary R. Lewin supervised the experiments, obtained funding and helped with surgeries. Julia Haseleu drafted the paper, with the help from Gary R. Lewin All authors approved the final version of the manuscript and agree to be accountable for all aspects of the work in ensuring that questions related to the accuracy or integrity of any part of the work are appropriately investigated and resolved. All persons designated as authors qualify for authorship, and all those who qualify for authorship are listed.

## CONFLICT OF INTEREST

None declared.

## Supporting information




**Supplementary Video S1**. Processing of tiled image stacks of CTB‐labelled spinal terminal fields. (a) Raw tiled image stack taken through a TDE‐cleared spinal cord. Two channels were recorded to collect both CTB fluorescence (shown) and autofluorescence (not shown). (b) By subtracting the autofluorescence channel from the CTB channel, autofluorescence was removed. (c) Using stack histogram‐based thresholding, the images were binarized. (d) Noise was eliminated by removing single voxels. (e) A summed dorsoventral projection was constructed to aid visualization and analysis. (f) The ImageJ colour look‐up table ‘16 Colors’ was applied to the summed dorsoventral projection. Scale bar: 150 µm. Abbreviation: LUT, look‐up table.

**Figure S1**. Response properties of muscle Aδ‐fibres newly innervating the skin compared to intact and regenerated cutaneous afferents in C57BL/6 mice.

**Figure S2**. Response properties of muscle Aδ‐fibres newly innervating the skin compared to intact and regenerated cutaneous afferents in *stoml3* mutant mice.

**Figure S3**. Volumetric imaging of CTB‐labelled afferent terminals in the spinal cord dorsal horn.

**Figure S4**. Control experiments ensuring reliable CTB injection performance.

**Figure S5**. Density measurements of spinal terminal fields in *stoml3* mutant and control mice.

**Figure S6**. Peripheral nerves after intraneural CTB‐injection into the cross anastomosed gastrocnemius nerve innervating the skin.

## Data Availability

The raw data in the paper is available on request.

## References

[eph13802-bib-0001] Aoyagi, Y. , Kawakami, R. , Osanai, H. , Hibi, T. , & Nemoto, T. (2015). A rapid optical clearing protocol using 2,2’‐thiodiethanol for microscopic observation of fixed mouse brain. PLoS ONE, 10(1), e0116280. 25633541 10.1371/journal.pone.0116280 PMC4310605

[eph13802-bib-0002] Becker, K. , Jährling, N. , Saghafi, S. , & Dodt, H.‐U. (2013). Dehydration and clearing of whole mouse brains and dissected hippocampi for ultramicroscopy. Cold Spring Harbor protocols, 2013, 683–684. 23818673 10.1101/pdb.prot075820

[eph13802-bib-0003] Beggs, S. , Torsney, C. , Drew, L. J. , & Fitzgerald, M. (2002). The postnatal reorganization of primary afferent input and dorsal horn cell receptive fields in the rat spinal cord is an activity‐dependent process. European Journal of Neuroscience, 16(7), 1249–1258. 12405985 10.1046/j.1460-9568.2002.02185.x

[eph13802-bib-0004] Bernal Sierra, Y. A. , Haseleu, J. , Kozlenkov, A. , Bégay, V. , & Lewin, G. R. (2017). Genetic tracing of Cav3.2 T‐type calcium channel expression in the peripheral nervous system. Frontiers in Molecular Neuroscience, 10, 70. 28360836 10.3389/fnmol.2017.00070 PMC5350092

[eph13802-bib-0005] Brown, A. G. (1981). Organization in the Spinal Cord: The Anatomy and Physiology of Identified Neurones. Springer.

[eph13802-bib-0006] Brown, A. G. , & Fyffe, R. E. (1978). The morphology of group Ia afferent fibre collaterals in the spinal cord of the cat. The Journal of Physiology, 274(1), 111–127. 624988 10.1113/jphysiol.1978.sp012137 PMC1282480

[eph13802-bib-0007] Brown, A. G. , & Fyffe, R. E. (1979). The morphology of group Ib afferent fibre collaterals in the spinal cord of the cat. The Journal of Physiology, 296(1), 215–226. 529088 10.1113/jphysiol.1979.sp013001 PMC1279074

[eph13802-bib-0008] Burgess, P. R. , & Horch, K. W. (1973). Specific regeneration of cutaneous fibers in the cat. Journal of Neurophysiology, 36(1), 101–114. 4705663 10.1152/jn.1973.36.1.101

[eph13802-bib-0009] Chakrabarti, S. , Klich, J. D. , Khallaf, M. A. , Hulme, A. J. , Sánchez‐Carranza, O. , Baran, Z. M. , Rossi, A. , Huang, A. T.‐L. , Pohl, T. , Fleischer, R. , Fürst, C. , Hammes, A. , Bégay, V. , Hörnberg, H. , Finol‐Urdaneta, R. K. , Poole, K. , Dottori, M. , & Lewin, G. R. (2024). Touch sensation requires the mechanically gated ion channel ELKIN1. Science, 383(6686), 992–998. 38422143 10.1126/science.adl0495

[eph13802-bib-0010] Chen, Z.‐L. , Yu, W.‐M. , & Strickland, S. (2007). Peripheral regeneration. Annual Review of Neuroscience, 30(1), 209–233. 10.1146/annurev.neuro.30.051606.094337 17341159

[eph13802-bib-0011] Chesler, A. T. , Szczot, M. , Bharucha‐Goebel, D. , Čeko, M. , Donkervoort, S. , Laubacher, C. , Hayes, L. H. , Alter, K. , Zampieri, C. , Stanley, C. , Innes, A. M. , Mah, J. K. , Grosmann, C. M. , Bradley, N. , Nguyen, D. , Foley, A. R. , Le Pichon, C. E. , & Bönnemann, C. G. (2016). The Role of PIEZO2 in Human Mechanosensation. The New England Journal of Medicine, 375(14), 1355–1364. 27653382 10.1056/NEJMoa1602812 PMC5911918

[eph13802-bib-0012] Cho, Y. , Shin, J. E. , Ewan, E. E. , Oh, Y. M. , Pita‐Thomas, W. , & Cavalli, V. (2015). Activating injury‐responsive genes with hypoxia enhances axon regeneration through neuronal HIF‐1α. Neuron, 88(4), 720–734. 26526390 10.1016/j.neuron.2015.09.050 PMC4655162

[eph13802-bib-0013] Costantini, I. , Ghobril, J.‐P. , Di Giovanna, A. P. , Allegra Mascaro, A. L. , Silvestri, L. , Müllenbroich, M. C. , Onofri, L. , Conti, V. , Vanzi, F. , Sacconi, L. , Guerrini, R. , Markram, H. , Iannello, G. , & Pavone, F. S. (2015). A versatile clearing agent for multi‐modal brain imaging. Scientific Reports, 5(1), 9808. 25950610 10.1038/srep09808 PMC4423470

[eph13802-bib-0014] Desiderio, S. , Schwaller, F. , Tartour, K. , Padmanabhan, K. , Lewin, G. R. , Carroll, P. , & Marmigere, F. (2024). Touch receptor end‐organ innervation and function require sensory neuron expression of the transcription factor Meis2 ed. VijayRaghavan K. eLife, 12, RP89287. 38386003 10.7554/eLife.89287 PMC10942617

[eph13802-bib-0015] Dykes, R. W. , & Terzis, J. K. (1979). Reinnervation of glabrous skin in baboons: Properties of cutaneous mechanoreceptors subsequent to nerve crush. Journal of Neurophysiology, 42(5), 1461–1478. 114613 10.1152/jn.1979.42.5.1461

[eph13802-bib-0016] Ertürk, A. , Becker, K. , Jährling, N. , Mauch, C. P. , Hojer, C. D. , Egen, J. G. , Hellal, F. , Bradke, F. , Sheng, M. , & Dodt, H.‐U. (2012). Three‐dimensional imaging of solvent‐cleared organs using 3DISCO. Nature Protocols, 7(11), 1983–1995. 23060243 10.1038/nprot.2012.119

[eph13802-bib-0017] Ertürk, A. , Mauch, C. P. , Hellal, F. , Förstner, F. , Keck, T. , Becker, K. , Jährling, N. , Steffens, H. , Richter, M. , Hübener, M. , Kramer, E. , Kirchhoff, F. , Dodt, H. U. , & Bradke, F. (2011). Three‐dimensional imaging of the unsectioned adult spinal cord to assess axon regeneration and glial responses after injury. Nature Medicine, 18(1), 166–171. 10.1038/nm.2600 22198277

[eph13802-bib-0018] Fawcett, J. W. , & Keynes, R. J. (1990). Peripheral Nerve Regeneration. Annual Review of Neuroscience, 13(1), 43–60. 10.1146/annurev.ne.13.030190.000355 2183684

[eph13802-bib-0019] Granmo, M. , Petersson, P. , & Schouenborg, J. (2008). Action‐based body maps in the spinal cord emerge from a transitory floating organization. Journal of Neuroscience, 28(21), 5494–5503. 18495883 10.1523/JNEUROSCI.0651-08.2008 PMC6670632

[eph13802-bib-0020] Hoheisel, U. , Lehmann‐Willenbrock, E. , & Mense, S. (1989). Termination patterns of identified group II and III afferent fibres from deep tissues in the spinal cord of the cat. Neuroscience, 28(2), 495–507. 2522168 10.1016/0306-4522(89)90195-4

[eph13802-bib-0021] Horch, K. W. , & Lisney, S. J. (1981). On the number and nature of regenerating myelinated axons after lesions of cutaneous nerves in the cat. The Journal of Physiology, 313(1), 275–286. 7277219 10.1113/jphysiol.1981.sp013664 PMC1274450

[eph13802-bib-0022] Horch, K. (1979). Guidance of regrowing sensory axons after cutaneous nerve lesions in the cat. Journal of Neurophysiology, 42(5), 1437–1449. 490201 10.1152/jn.1979.42.5.1437

[eph13802-bib-0023] Johnson, R. D. , Taylor, J. S. , Mendell, L. M. , & Munson, J. B. (1995). Rescue of motoneuron and muscle afferent function in cats by regeneration into skin. I. Properties of afferents. Journal of Neurophysiology, 73(2), 651–661. 7760124 10.1152/jn.1995.73.2.651

[eph13802-bib-0024] Kloepper, J. E. , Bíró, T. , Paus, R. , & Cseresnyés, Z. (2010). Point scanning confocal microscopy facilitates 3D human hair follicle imaging in tissue sections. Experimental Dermatology, 19(7), 691–694. 20545762 10.1111/j.1600-0625.2010.01110.x

[eph13802-bib-0025] Koerber, H. R. , Seymour, A. W. , & Mendell, L. M. (1989). Mismatches between peripheral receptor type and central projections after peripheral nerve regeneration. Neuroscience Letters, 99(1‐2), 67–72. 2748020 10.1016/0304-3940(89)90266-8

[eph13802-bib-0026] Korsching, S. , & Thoenen, H. (1983). Nerve growth factor in sympathetic ganglia and corresponding target organs of the rat: Correlation with density of sympathetic innervation. Proceedings of the National Academy of Sciences of the United States of America, 80(11), 3513–3516. 6407016 10.1073/pnas.80.11.3513 PMC394075

[eph13802-bib-0027] Koschorke, G. M. , Meyer, R. A. , & Campbell, J. N. (1994). Cellular components necessary for mechanoelectrical transduction are conveyed to primary afferent terminals by fast axonal transport. Brain Research, 641(1), 99–104. 8019856 10.1016/0006-8993(94)91820-1

[eph13802-bib-0028] Lechner, S. G. , & Lewin, G. R. (2013). Hairy Sensation. Physiology, 28(3), 142–150. 23636260 10.1152/physiol.00059.2012

[eph13802-bib-0029] Lewin, G. R. , McKintosh, E. , & McMahon, S. B. (1994). NMDA receptors and activity‐dependent tuning of the receptive fields of spinal cord neurons. Nature, 369(6480), 482–485. 8202138 10.1038/369482a0

[eph13802-bib-0030] Lewin, G. R. , & McMahon, S. B. (1991a). Physiological properties of primary sensory neurons appropriately and inappropriately innervating skin in the adult rat. Journal of Neurophysiology, 66(4), 1205–1217. 1761980 10.1152/jn.1991.66.4.1205

[eph13802-bib-0031] Lewin, G. R. , & McMahon, S. B. (1991b). Physiological properties of primary sensory neurons appropriately and inappropriately innervating skeletal muscle in adult rats. Journal of Neurophysiology, 66(4), 1218–1231. 1761981 10.1152/jn.1991.66.4.1218

[eph13802-bib-0032] Lewin, G. R. , & McMahon, S. B. (1993). Muscle afferents innervating skin form somatotopically appropriate connections in the adult rat dorsal horn. European Journal of Neuroscience, 5(8), 1083–1092. 8281312 10.1111/j.1460-9568.1993.tb00962.x

[eph13802-bib-0033] Lewin, G. R. , Winter, J. , & McMahon, S. B. (1992). Regulation of afferent connectivity in the adult spinal cord by nerve growth factor. European Journal of Neuroscience, 4(8), 700–707. 12106314 10.1111/j.1460-9568.1992.tb00179.x

[eph13802-bib-0034] Mahar, M. , & Cavalli, V. (2018). Intrinsic mechanisms of neuronal axon regeneration. Nature Reviews Neuroscience, 19(6), 323–337. 29666508 10.1038/s41583-018-0001-8 PMC5987780

[eph13802-bib-0035] Maksimovic, S. , Nakatani, M. , Baba, Y. , Nelson, A. M. , Marshall, K. L. , Wellnitz, S. A. , Firozi, P. , Woo, S.‐H. , Ranade, S. , Patapoutian, A. , & Lumpkin, E. A. (2014). Epidermal Merkel cells are mechanosensory cells that tune mammalian touch receptors. Nature, 509(7502), 617–621. 24717432 10.1038/nature13250 PMC4097312

[eph13802-bib-0066] McMahon, S. B. , & Wall, P. D. (1989). Changes in spinal cord reflexes after cross‐anastomosis of cutaneous and muscle nerves in the adult rat. Nature, 342(6247), 272–274. 2812026 10.1038/342272a0

[eph13802-bib-0036] McMahon, S. B. , & Gibson, S. (1987). Peptide expression is altered when afferent nerves reinnervate inappropriate tissue. Neuroscience letters, 73(1), 9–15. 2436099 10.1016/0304-3940(87)90022-x

[eph13802-bib-0037] Mense, S. (1996). Group III and IV receptors in skeletal muscle: Are they specific or polymodal? Progress in Brain Research, 113, 83–100. 9009729 10.1016/s0079-6123(08)61082-1

[eph13802-bib-0038] Molander, C. , & Grant, G. (1987). Spinal cord projections from hindlimb muscle nerves in the rat studied by transganglionic transport of horseradish peroxidase, wheat germ agglutinin conjugated horseradish peroxidase, or horseradish peroxidase with dimethylsulfoxide. Journal of Comparative Neurology, 260(2), 246–255. 3038969 10.1002/cne.902600208

[eph13802-bib-0039] Moshourab, R. A. , Wetzel, C. , Martinez‐Salgado, C. , & Lewin, G. R. (2013). Stomatin‐domain protein interactions with acid‐sensing ion channels modulate nociceptor mechanosensitivity. The Journal of Physiology, 591(22), 5555–5574. 23959680 10.1113/jphysiol.2013.261180 PMC3853495

[eph13802-bib-0040] Ojeda‐Alonso, J. , Calvo‐Enrique, L. , Paricio‐Montesinos, R. , Kumar, R. , Zhang, M.‐D. , Poulet, J. F. A. , Ernfors, P. , & Lewin, G. R. (2024). Sensory Schwann cells set perceptual thresholds for touch and selectively regulate mechanical nociception. Nature Communications, 15(1), 898. 10.1038/s41467-024-44845-8 PMC10847425 38320986

[eph13802-bib-0041] Ollion, J. , Cochennec, J. , Loll, F. , Escudé, C. , & Boudier, T. (2013). TANGO: A generic tool for high‐throughput 3D image analysis for studying nuclear organization. Bioinformatics (Oxford, England), 29, 1840–1841. 23681123 10.1093/bioinformatics/btt276 PMC3702251

[eph13802-bib-0042] Panneton, W. M. , Gan, Q. , & Juric, R. (2005). The central termination of sensory fibers from nerves to the gastrocnemius muscle of the rat. Neuroscience, 134(1), 175–187. 15953682 10.1016/j.neuroscience.2005.02.032

[eph13802-bib-0043] Poole, K. , Herget, R. , Lapatsina, L. , Ngo, H.‐D. , & Lewin, G. R. (2014). Tuning Piezo ion channels to detect molecular‐scale movements relevant for fine touch. Nature Communications, 5(1), 3520. 10.1038/ncomms4520 PMC3973071 24662763

[eph13802-bib-0044] Preibisch, S. , Saalfeld, S. , & Tomancak, P. (2009). Globally optimal stitching of tiled 3D microscopic image acquisitions. Bioinformatics, 25(11), 1463–1465. 19346324 10.1093/bioinformatics/btp184 PMC2682522

[eph13802-bib-0045] Ranade, S. S. , Woo, S.‐H. , Dubin, A. E. , Moshourab, R. A. , Wetzel, C. , Petrus, M. , Mathur, J. , Bégay, V. , Coste, B. , Mainquist, J. , Wilson, A. J. , Francisco, A. G. , Reddy, K. , Qiu, Z. , Wood, J. N. , Lewin, G. R. , & Patapoutian, A. (2014). Piezo2 is the major transducer of mechanical forces for touch sensation in mice. Nature, 516(7529), 121–125. 25471886 10.1038/nature13980 PMC4380172

[eph13802-bib-0046] Rbia, N. , & Shin, A. Y. (2017). The role of nerve graft substitutes in motor and mixed motor/sensory peripheral nerve injuries. The Journal of Hand Surgery, 42(5), 367–377. 28473159 10.1016/j.jhsa.2017.02.017

[eph13802-bib-0047] Rivers, W. H. R. , & Head, H. (1908). A human experiment in nerve division. Brain, 31(3), 323–450. 10.1093/brain/awp288 19877308

[eph13802-bib-0048] Robertson, B. , & Arvidsson, J. (1985). Transganglionic transport of wheat germ agglutinin‐HRP and choleragenoid‐HRP in rat trigeminal primary sensory neurons. Brain Research, 348(1), 44–51. 2415219 10.1016/0006-8993(85)90357-9

[eph13802-bib-0049] Sánchez‐Carranza, O. , Chakrabarti, S. , Kühnemund, J. , Schwaller, F. , Bégay, V. , García‐Contreras, J. A. , Wang, L. , & Lewin, G. R. (2024). Piezo2 voltage‐block regulates mechanical pain sensitivity. Brain, 147(10), 3487–3500. 38984717 10.1093/brain/awae227 PMC11449130

[eph13802-bib-0050] Schneider, C. A. , Rasband, W. S. , & Eliceiri, K. W. (2012). NIH Image to ImageJ: 25 years of image analysis. Nature Methods, 9(7), 671–675. 22930834 10.1038/nmeth.2089 PMC5554542

[eph13802-bib-0051] Shelton, D. L. , & Reichardt, L. F. (1984). Expression of the beta‐nerve growth factor gene correlates with the density of sympathetic innervation in effector organs. Proceedings of the National Academy of Sciences of the United States of America, 81(24), 7951–7955. 6595669 10.1073/pnas.81.24.7951 PMC392271

[eph13802-bib-0052] Shin, J. B. , Martinez‐Salgado, C. , Heppenstall, P. A. , & Lewin, G. R. (2003). A T‐type calcium channel required for normal function of a mammalian mechanoreceptor. Nature Neuroscience, 6(7), 724–730. 12808460 10.1038/nn1076

[eph13802-bib-0053] Shortland, P. , & Woolf, C. J. (1993). Morphology and somatotopy of the central arborizations of rapidly adapting glabrous skin afferents in the rat lumbar spinal cord. Journal of Comparative Neurology, 329(4), 491–511. 8454737 10.1002/cne.903290406

[eph13802-bib-0054] Shortland, P. , Woolf, C. J. , & Fitzgerald, M. (1989). Morphology and somatotopic organization of the central terminals of hindlimb hair follicle afferents in the rat lumbar spinal cord. Journal of Comparative Neurology, 289(3), 416–433. 2808777 10.1002/cne.902890307

[eph13802-bib-0055] Song, Y. , Li, D. , Farrelly, O. , Miles, L. , Li, F. , Kim, S. E. , Lo, T. Y. , Wang, F. , Li, T. , Thompson‐Peer, K. L. , Gong, J. , Murthy, S. E. , Coste, B. , Yakubovich, N. , Patapoutian, A. , Xiang, Y. , Rompolas, P. , Jan, L. Y. , & Jan, Y. N. (2019). The mechanosensitive ion channel piezo inhibits axon regeneration. Neuron, 102(2), 373–389.e6.e6. 30819546 10.1016/j.neuron.2019.01.050 PMC6487666

[eph13802-bib-0056] Staudt, T. , Lang, M. C. , Medda, R. , Engelhardt, J. , & Hell, S. W. (2007). 2,2’‐thiodiethanol: A new water soluble mounting medium for high resolution optical microscopy. Microscopy Research and Technique, 70(1), 1–9. 17131355 10.1002/jemt.20396

[eph13802-bib-0057] Tedeschi, A. , & Bradke, F. (2017). Spatial and temporal arrangement of neuronal intrinsic and extrinsic mechanisms controlling axon regeneration. Current Opinion in Neurobiology, 42, 118–127. 28039763 10.1016/j.conb.2016.12.005

[eph13802-bib-0058] Terzis, J. K. , & Dykes, R. W. (1980). Reinnervation of glabrous skin in baboons: Properties of cutaneous mechanoreceptors subsequent to nerve transection. Journal of Neurophysiology, 44(6), 1214–1225. 7452327 10.1152/jn.1980.44.6.1214

[eph13802-bib-0059] Tröster, P. , Haseleu, J. , Petersen, J. , Drees, O. , Schmidtko, A. , Schwaller, F. , Lewin, G. R. , Ter‐Avetisyan, G. , Winter, Y. , Peters, S. , Feil, S. , Feil, R. , Rathjen, F. G. , & Schmidt, H. (2018). The absence of sensory axon bifurcation affects nociception and termination fields of afferents in the spinal cord. Frontiers in Molecular Neuroscience, 11, 19. 29472841 10.3389/fnmol.2018.00019 PMC5809486

[eph13802-bib-0060] Walcher, J. , Ojeda‐Alonso, J. , Haseleu, J. , Oosthuizen, M. K. , Rowe, A. H. , Bennett, N. C. , & Lewin, G. R. (2018). Specialized mechanoreceptor systems in rodent glabrous skin. The Journal of Physiology, 596(20), 4995–5016. 30132906 10.1113/JP276608 PMC6187043

[eph13802-bib-0061] Wan, X. C. , Trojanowski, J. Q. , & Gonatas, J. O. (1982). Cholera toxin and wheat germ agglutinin conjugates as neuroanatomical probes: Their uptake and clearance, transganglionic and retrograde transport and sensitivity. Brain Research, 243(2), 215–224. 6179573 10.1016/0006-8993(82)90244-x

[eph13802-bib-0062] Wang, R. , & Lewin, G. R. (2011). The Cav3.2 T‐type calcium channel regulates temporal coding in mouse mechanoreceptors. The Journal of Physiology, 589(9), 2229–2243. 21486775 10.1113/jphysiol.2010.203463 PMC3098700

[eph13802-bib-0063] Wetzel, C. , Hu, J. , Riethmacher, D. , Benckendorff, A. , Harder, L. , Eilers, A. , Moshourab, R. , Kozlenkov, A. , Labuz, D. , Caspani, O. , Erdmann, B. , Machelska, H. , Heppenstall, P. A. , & Lewin, G. R. (2007). A stomatin‐domain protein essential for touch sensation in the mouse. Nature, 445(7124), 206–209. 17167420 10.1038/nature05394

[eph13802-bib-0064] Wetzel, C. , Pifferi, S. , Picci, C. , Gök, C. , Hoffmann, D. , Bali, K. K. , Lampe, A. , Lapatsina, L. , Fleischer, R. , Smith, E. S. , Bégay, V. , Moroni, M. , Estebanez, L. , Kühnemund, J. , Walcher, J. , Specker, E. , Neuenschwander, M. , von Kries, J. P. , Haucke, V. , … Lewin, G. R. (2017). Small‐molecule inhibition of STOML3 oligomerization reverses pathological mechanical hypersensitivity. Nature Neuroscience, 20(2), 209–218. 27941788 10.1038/nn.4454

[eph13802-bib-0065] Woo, S.‐H. , Lukacs, V. , de Nooij, J. C. , Zaytseva, D. , Criddle, C. R. , Francisco, A. , Jessell, T. M. , Wilkinson, K. A. , & Patapoutian, A. (2015). Piezo2 is the principal mechanotransduction channel for proprioception. Nature Neuroscience, 18(12), 1756–1762. 26551544 10.1038/nn.4162 PMC4661126

