# Supplementary figures and images for: The mechanotransduction protein STOML3 is required for proprioceptor plasticity following peripheral nerve regeneration
Source: Exp Physiol. 2025 Mar 31;110(10):1473–91. doi: 10.1113/EP092428 (PMC12486322; doi:10.1113/EP092428)

**a**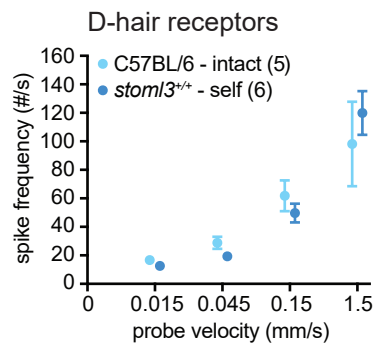**b**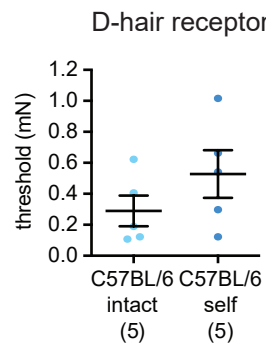**c**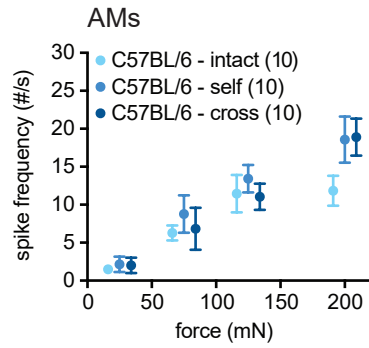**d**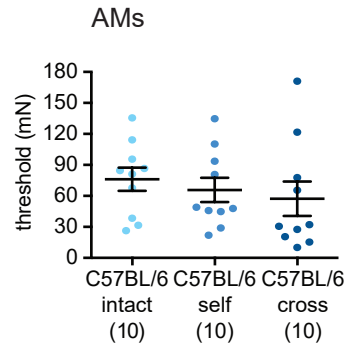

Supplement: Supplementary file 2 — Figure S1. Response properties of muscle Aδ‐fibres newly innervating the skin compared to intact and regenerated cutaneous afferents in C57BL/6 mice. [file EPH-110-1473-s006.pdf]

**e**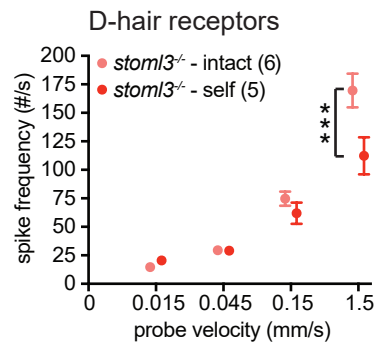**f**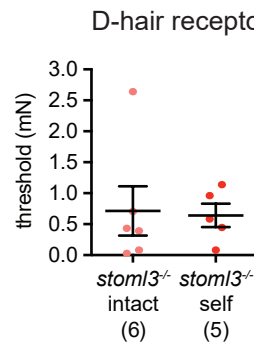**g**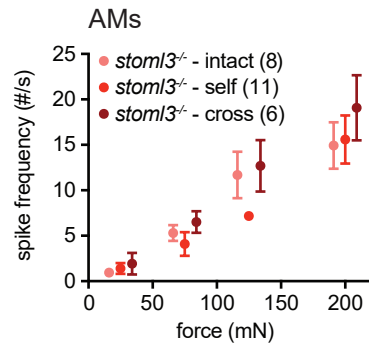**h**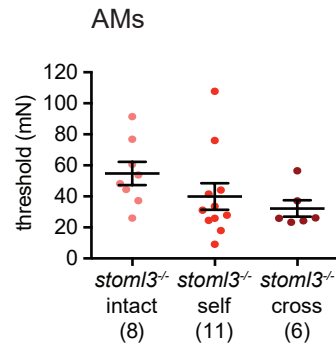

Supplement: Supplementary file 3 — Figure S2. Response properties of muscle Aδ‐fibres newly innervating the skin compared to intact and regenerated cutaneous afferents in stoml3 mutant mice. [file EPH-110-1473-s003.pdf]

**a**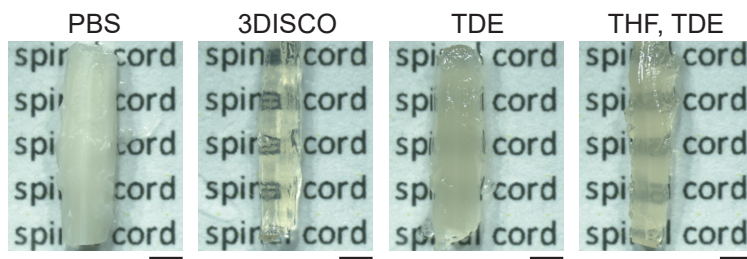**b**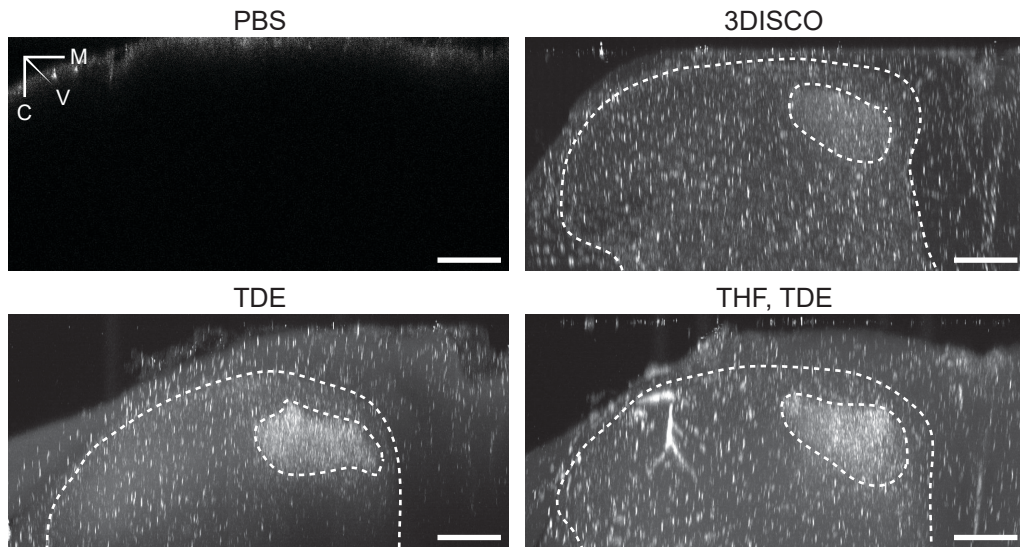

Supplement: Supplementary file 4 — Figure S3. Volumetric imaging of CTB‐labelled afferent terminals in the spinal cord dorsal horn. [file EPH-110-1473-s001.pdf]

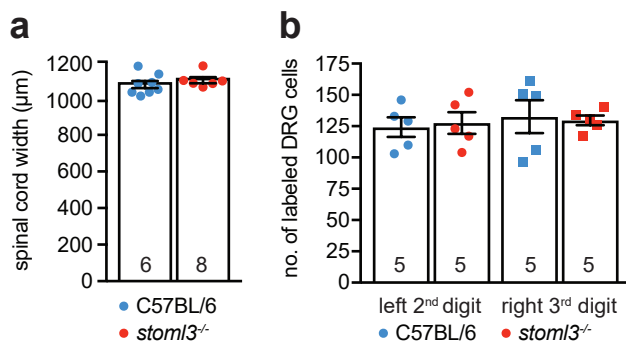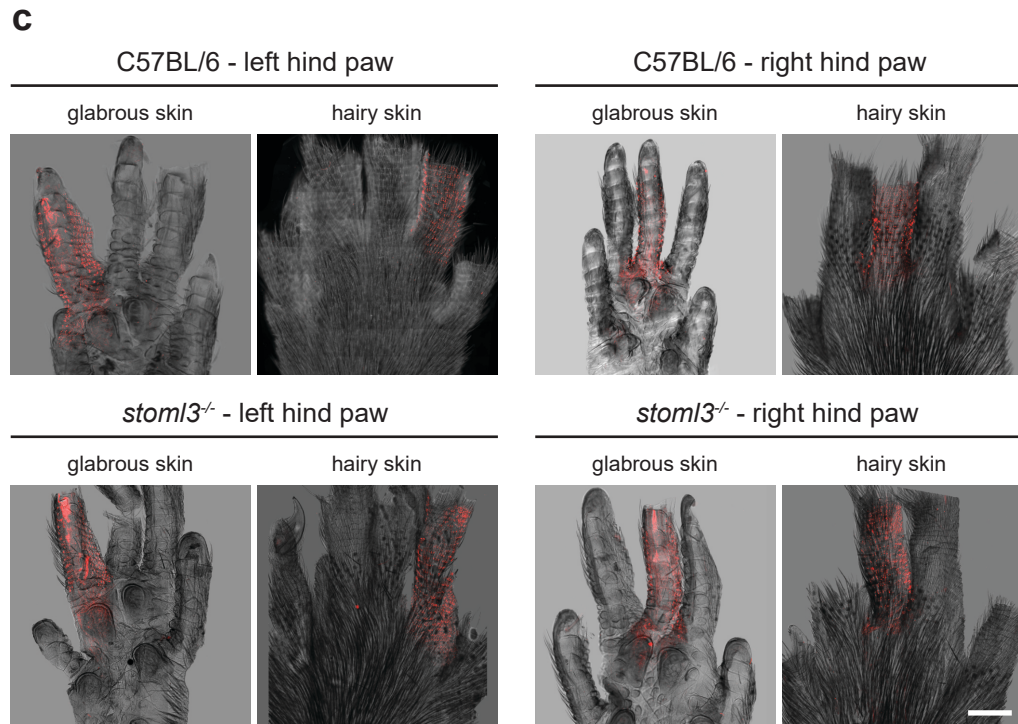

Supplement: Supplementary file 5 — Figure S4. Control experiments ensuring reliable CTB injection performance. [file EPH-110-1473-s002.pdf]

**a**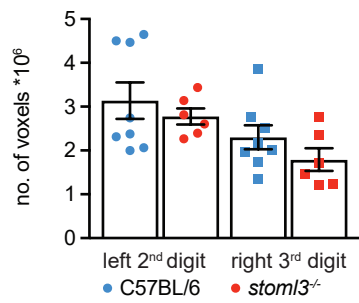**b**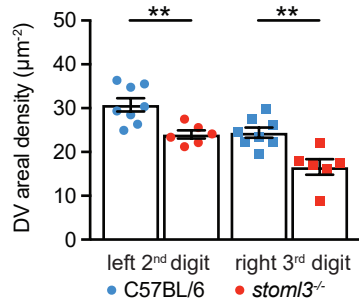**c**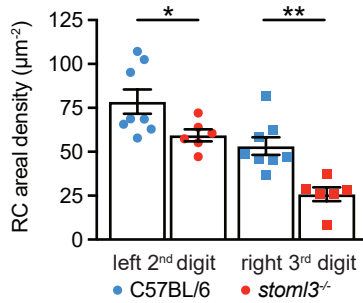**d**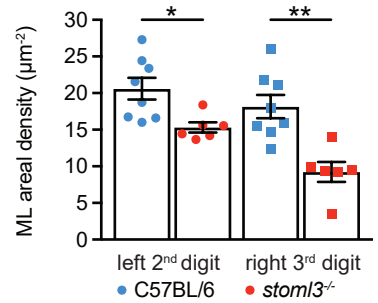**e**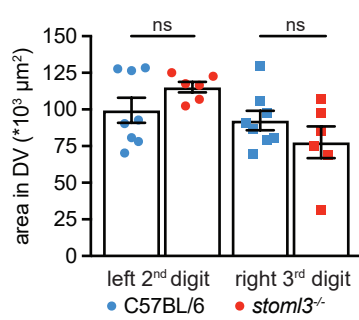**f**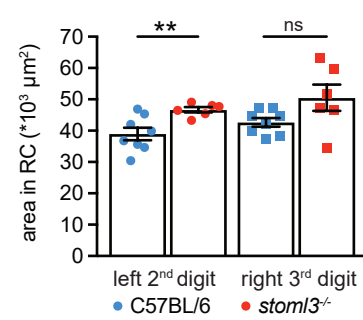**g**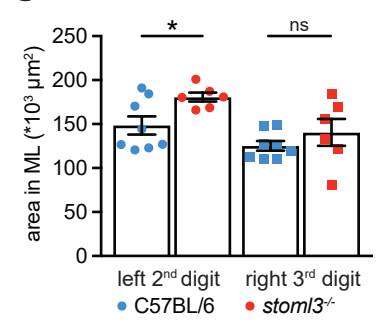

Supplement: Supplementary file 6 — Figure S5. Density measurements of spinal terminal fields in stoml3 mutant and control mice. [file EPH-110-1473-s005.pdf]

C57BL/6

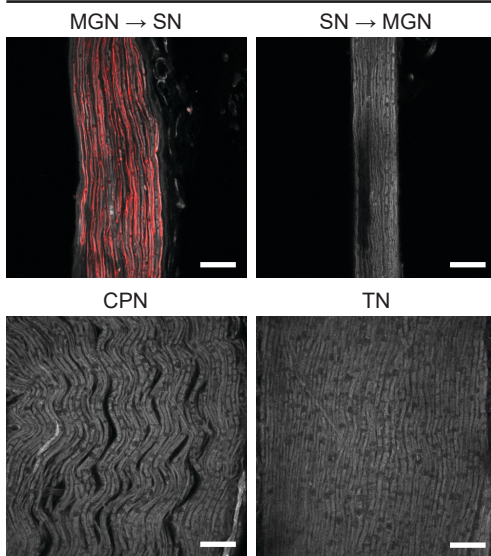

*stoml3*<sup>-/-</sup>

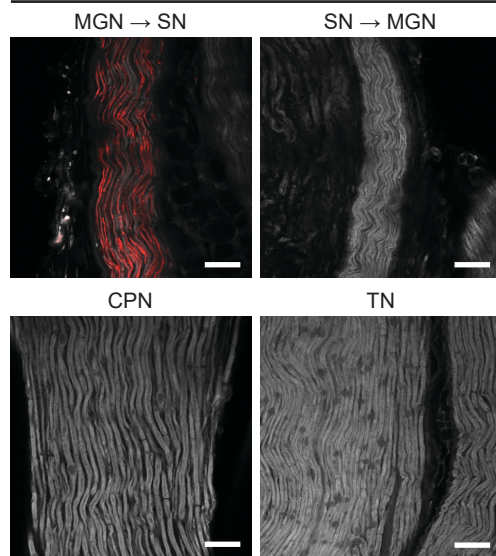

CTB-AL594 / autofluorescence

Supplement: Supplementary file 7 — Figure S6. Peripheral nerves after intraneural CTB‐injection into the cross anastomosed gastrocnemius nerve innervating the skin. [file EPH-110-1473-s007.pdf]
